# Revisiting imperfect interface laws for two-dimensional elastodynamics

**DOI:** 10.1098/rspa.2020.0519

**Published:** 2021-01-13

**Authors:** Kim Pham, Agnès Maurel, Jean-Jacques Marigo

**Affiliations:** 1 IMSIA, CNRS, EDF, CEA, ENSTA Paris, Institut Polytechnique de Paris, 828 Bd des Maréchaux, 91732 Palaiseau, France; 2 Institut Langevin, ESPCI Paris, Université PSL, CNRS, 1 rue Jussieu, Paris 75005, France; 3 Laboratoire de Mécanique des Solides, CNRS, Ecole Polytechnique, 91120 Palaiseau, France

**Keywords:** elastodynamics, imperfect interface, periodic defects, void/crack, high-order homogenization, asymptotic analysis

## Abstract

We study the interaction of in-plane elastic waves with imperfect interfaces composed of a periodic array of voids or cracks. An effective model is derived from high-order asymptotic analysis based on two-scale homogenization and matched asymptotic technique. In two-dimensional elasticity, we obtain jump conditions set on the in-plane displacements and normal stresses; the jumps involve in addition effective parameters provided by static, elementary problems being the equivalents of the cell problems in classical two-scale homogenization. The derivation of the model is conducted in the transient regime and its stability is guarantied by the positiveness of the effective interfacial energy. Spring models are envisioned as particular cases. It is shown that *massless-spring* models are recovered in the limit of small void thicknesses and collinear cracks. By contrast, the use of *mass-spring* model is justified at normal incidence, otherwise unjustified. We provide quantitative validations of our model and comparison with spring models by means of comparison with direct numerical calculations in the harmonic regime.

## 1. Introduction

Surfaces of separations between two elastic solids can impact significantly the propagation of waves. In 1967, Jones & Whittier [1] inspected the ability of thin elastic bonds to support guided waves. Considering inertialess layers with increasing stiffness, they exhibited a family of guided waves with limiting cases being the Rayleigh waves and the Stoneley waves. Nine years later, Murty [2] considered the scattering by what he called *loosely bonded interfaces*; having in mind situations as different as aligned cracks or thin viscous layers, he postulated the simplest contact law able to recover a perfect interface and a fully debonded interface as limiting cases. Assuming that the normal stress *σ* remains unconditionally continuous across the imperfect interface, his model coincides with the *massless-spring model* of [1]: in a scalar case, it reads $\sigma =\kappa [[u]]\;\text{with}\;[[u]]=(u^{+}-u^{-})$ the jump of the displacement *u* across the interface and *κ* a parameter termed bonding parameter or stiffness. Later on, Baik and Thompson have enriched the model by including inertial terms; the resulting *mass-spring model* imposes displacements and normal stresses discontinuities [3]. A review of the different models has been analysed by Martin [4] who showed that some of them suffer from non-uniqueness.

The case of thin homogeneous layers is of interest for practical applications; besides the exact solution is available. In a series of papers, Rokhlin and co-workers have developed accurate transmission conditions and inspected the validity of spring models [5–7]. This has revealed the limited range of validity of the massless-spring model; in particular, neglecting the inertial terms appears to be unjustified [8–10]. The case of thin layer interfaces containing cracks, micropores, voids or faults has also received attention in particular for applications in seismology and in engineering for non-destructive testing. Their academic study started in the 1980s with the works of Achenbach and his co-workers who investigated the scattering properties in many situations including equally spaced collinear cracks [11,12], inclined cracks [13], spherical cavities [14] as well as randomly distributed cracks [15]; a review is presented in [16]. Exact solutions are in general not available and numerical solutions have been sought by means of multiple scattering theory and boundary integral equation method. For zero thickness cracks, the validity of the spring models has been demonstrated [11,12,17–19]. However, a quantitative disagreement for crack like defects, or diffusion bonds, with small but finite thickness has been reported in [19].

The present works aims to derive a model on the basis of rational approximations for defects with non-zero thickness and to assess if the use of spring models is legitimate in some limit. To that aim, we extend the results of Marigo and co-workers [20,21] developed in a static context; the idea is to combine two-scale homogenization theory to treat the periodicity of the inhomogeneities and matched asymptotic technique to deal with the thin layer. In §2, we introduce the full-scale problem of a periodic array of voids and the effective problem whose derivation is detailed in §3. Simplifications of our model for waves at normal incidence and for vanishing thickness of the cracks are envisioned, see §3e. It is shown that for very thin voids, which means with a thickness much smaller than the array spacing, a *massless-spring model* is obtained which involves two different, tangential and normal, stiffnesses. The model further simplifies for cracks with zero thickness, resulting in a single stiffness whose form is explicit, and conform with the model of Angel & Achenbach [11]. Amusingly, and contrarily to the case of a thin homogeneous layer, keeping in addition an inertial term is unjustified except at normal incidence. Hence the *mass-spring model* does not apply in general to an array of thin voids (and it does not apply to cracks which are inertialess by construction). Our analysis is set in the time domain which allows us to analyse the energetic properties of the effective problem, see §4. The jump conditions produce a non classical contribution to the flux of the elastic Poynting vector. It is shown that this contribution reads as the time derivative of a positive effective energy which guaranties the stability in the transient regime. Section 5 illustrates the effectiveness of the our model by comparison with full-scale simulations of the two-dimensional elastic problem [22]. (Closed-forms of the scattering coefficients and of the fields are provided.) We take the opportunity in this section to discuss the effectiveness of other formulations (zero-thickness formulation and massless-spring models). We provide in §6 concluding remarks and extensions of the study.

## 2. The actual problem and the effective problem

**Figure 1.**
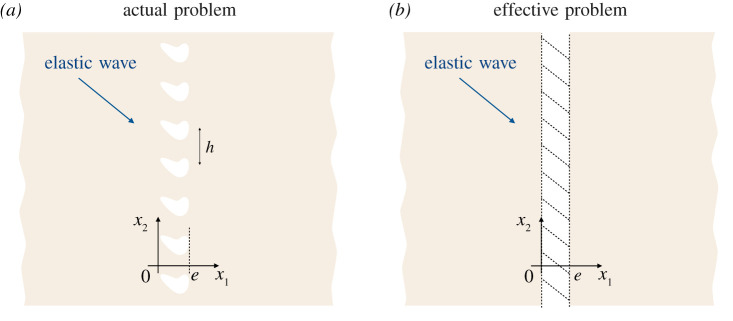
The problem of in-plane elastic waves interacting with a periodic array of voids embedded in a matrix of Lamé coefficients (*λ*, *μ*) and mass density *ρ*. (*a*) The actual problem and (*b*) the effective problem (after homogenization); the effect of the array of voids is captured by the jump conditions (2.2) applied between *x*_1_ = 0 and *x*_1_ = *e*. (Online version in colour.)

We consider a two-dimensional linear elasticity problem where a periodic array of voids, identical but of arbitrary shape, is embedded in a homogeneous matrix of Lamé coefficients (*λ*, *μ*) and mass density *ρ*, figure 1. With ***x*** = (*x*_1_, *x*_2_) the coordinate system, the voids are evenly distributed along *x*_2_ and within *x*_1_ ∈ (0, *e*) with a spacing *h* and *e* is comparable to *h*. We consider the propagation of elastic waves in the time domain, with *t* the time. Denoting (***u***, **σ**, **ε**_***x***_) the displacement vector, the stress and the strain tensors, the governing equations in the actual problem read

2.1 $${\text{div}}_{x}\sigma =\rho \ddot{u},\;\sigma =\lambda \text{tr}(\varepsilon_{x}(u))1+2\mu \varepsilon_{x}(u),\;\text{with }\varepsilon_{x}(u)=\frac{1}{2}(\nabla_{x}u+{\;}^{t}\nabla_{x}u),$$

where dot means time derivative and **1** stands for the identity matrix. The above problem is complemented with stress-free conditions **σn** = **0** on the boundary of the voids and radiation conditions of the Sommerfeld type. (At this stage, we do not need to specify them.) In the following, with (***e***_1_, ***e***_2_) the unit vectors of the basis, we denote *u*_*i*_ = ***u*** · ***e***_*i*_, *i* = 1, 2 and *σ*_*ij*_ = **σ** · ***e***_*i*_ ⊗ ***e***_*j*_, (*i*, *j*) ∈ {1, 2}^2^.

We consider a source which imposes a typical, or maximum, frequency *ω* such that *k*_T_ *h* ≪ 1 with *k*_T_ = *ω*/*c*_T_, $c_{\text{T}}=\omega \sqrt{\rho /\mu }$. In this subwavelength regime, our study aims to simplify the actual problem. Specifically, the separation of the length scales between the wavelength and the dimensions (*e*, *h*) of the array is exploited in an asymptotic analysis detailed in the forthcoming section. As a result, the array of voids is replaced by *jump conditions* across an effective interface with boundaries at *x*_1_ = 0 and *x*_1_ = *e*. (It is worth noticing that the region *x*_1_ ∈ (0, *e*) is not interrogated; only the values of the fields at *x*_1_ = 0 and *e* are concerned.) These jumps tell us that the in-plane displacements and the normal stress satisfy

2.2 $$[[u_{k}]]=(e\delta_{ik}\delta_{j1}+hB_{k}^{ij})\sum_{i,j}\bar{\frac{\partial u_{i}}{\partial x_{j}}},\;[[\sigma_{1k}]]=e(1-\varphi )\bar{\frac{\partial \sigma_{1k}}{\partial x_{1}}}-h\sum_{i,j}C_{k}^{ij}\frac{\partial \;}{\partial x_{2}}\bar{\frac{\partial u_{i}}{\partial x_{j}}},$$

with (*i*, *j*, *k*) ∈ {1, 2}^3^. In the above transmission conditions, for any scalar field *f*(***x***, *t*), we have defined

2.3 $$[[f]]=f(e,x_{2},t)-f(0,x_{2},t),\;\bar{f}=\frac{1}{2}(f(e,x_{2},t)+f(0,x_{2},t)),$$

being the jump and the mean value at the interface. The parameter φ is explicit, it is the area of a void rescaled with *he*. The parameters $\left(B_{k}^{ij},C_{k}^{ij}\right)$ are obtained from three elementary static problems depending only on the geometry of the voids. In general, (2.2) involves 10 non-zero effective parameters linked by four relations, see forthcoming (3.22) and (3.23), hence six parameters. Eventually for voids with symmetric shape with respect to *x*_1_ or to *x*_2_, only four effective parameters are involved.

## 3. Asymptotic analysis

As previously said, the asymptotic procedure is conducted owing to the separation of the length scales between the typical wavelength imposed by the source and the dimensions (*e*, *h*) of the array. We define

$$\eta =k_{\text{T}}h\ll 1$$

the small parameter measuring the separation of the length scales and *k*_T_ *e* = *O*(*η*). In this section, we use the dimensionless coordinate $x=(x_{1},x_{2})$ with $x=k_{\text{T}}x$. Next, for simplicity, we preserve the formulation (2.1) by choosing $t=k_{\text{T}}t$ and $u(x,t)=k_{\text{T}}u(x,t)$, σ(x,t)=σ(x,t). Doing so, the rescaled formulation of (2.1) reads

**Figure 2.**
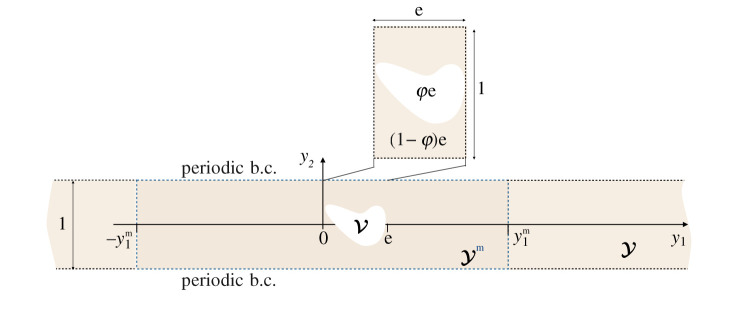
The inner problem is set in the elementary cell Y containing a single void with periodic boundary conditions at $y_{2}=\pm 1/2$. The void has a rescaled thickness e=e/h along $y_{1}$ and a rescaled surface area φe. (Online version in colour.)

3.1 $${\text{div}}_{\;}\sigma =\rho \ddot{}u,\;\sigma =\lambda \text{tr}(\varepsilon_{x}(u))1+2\mu \varepsilon_{x}(u),\;\text{with }\varepsilon_{x}(u)=\frac{1}{2}(\nabla_{x}u+{\;}^{t}\nabla_{x}u),$$

where dot means now derivative with respect to t. We also define the microscopic coordinate at the scale of the voids

$$\;y=\frac{x}{\eta },\;\;y\in Y=\left\{(y_{1},y_{2})\in R\times (-\frac{1}{2},\frac{1}{2})\right\}/V,$$

where V is the rescaled region of a single void (figure 2). We shall now define different expansions of the solution depending on either we are far or close to the array. Far from the array, the spatial variations of the fields are slow, being attributable to the wave propagation, hence to the large-scale 1/*k*_T_. Accordingly, we postulate asymptotic expansions for the *outer* solution of the form

3.2 $$u=\sum_{i=0}^{+\infty }\eta^{i}u^{i}(x,t)\;\text{and}\;\sigma =\sum_{i=0}^{+\infty }\eta^{i}\sigma^{i}(x,t).$$

In contrast close to the array, the fields have slow variations along $x_{2}$ due to phase variations of the waves along the array but they have also fast variations due to the evanescent field excited in the vicinity of the voids. (The evanescent field has spatial variations on the scale of *h*.) Hence, we postulate two-scale asymptotic expansions for the *inner* solution of the form

3.3 $$u=\sum_{i=0}^{+\infty }\eta^{i}v^{i}(x_{2},\;y,t)\;\text{and}\;\sigma =\sum_{i=0}^{+\infty }\eta^{i}\tau^{i}(x_{2},\;y,t).$$

The fields (**v**^*i*^, **τ**^*i*^) are 1-periodic functions w.r.t. $y_{2}$ which is formally equivalent to Bloch–Floquet conditions in the limit of infinite wavelengths. The resulting inner problem is set in the elementary cell Y (figure 2). For convenience, we also define $Y^{\text{m}}=\{y_{1}\in (-y_{1}^{\text{m}},y_{1}^{\text{m}}),y_{2}\in (-\frac{1}{2},\frac{1}{2})\}/V$, and $Y^{\text{m}}\to Y$ when $y_{1}^{\text{m}}\to +\infty$. It is worth noting that the boundary condition **τ**^*i*^**n** = **0** on the boundary of the voids applies to the inner solution (3.3); however there are missing boundary conditions when $y_{1}\to \pm \infty$. Reversely, the radiation conditions for $x_{1}\to \pm \infty$ apply to the outer solution (3.3) but boundary conditions when $x_{1}\to 0^{\pm }$ are missing. (These conditions will provide the effective transmission conditions.) Missing conditions are provided simultaneously by matching conditions which tell us that the outer and inner solutions coincide in an intermediate region, typically in the transient region where the evanescent field becomes negligible. The matching conditions are obtained by replacing $x_{1}$ by $\eta y_{1}$ in (3.3) and passing to the limit $y_{1}\to \pm \infty$ in (3.3). Doing so, we get at the zero order

3.4 $$u^{0}(x_{1}=0^{\pm },x_{2},t)=\lim_{y_{1}\to \pm \infty }v^{0}(x_{2},\;y,t),\;\sigma^{0}(x_{1}=0^{\pm },x_{2},t)=\lim_{y_{1}\to \pm \infty }\tau^{0}(x_{2},\;y,t),$$

and at the first order

3.5 $$\begin{array}{rl} & u^{1}(x_{1}=0^{\pm },x_{2},t)=\lim_{y_{1}\to \pm \infty }\left(v^{1}(x_{2},\;y,t)-y_{1}\frac{\partial u^{0}}{\partial x_{1}}(x_{1}=0^{\pm },x_{2},t)\right)\\ \;\text{and}\; & \sigma^{1}(x_{1}=0^{\pm },x_{2},t)=\lim_{y_{1}\to \pm \infty }\left(\tau^{1}(x_{2},\;y,t)-y_{1}\frac{\partial \sigma^{0}}{\partial x_{1}}(x_{1}=0^{\pm },x_{2},t)\right). \end{array}\}$$

It remains to establish the hierarchy of problems issued from (3.1) and satisfied by the terms of the outer and inner expansions. Given the form of the expansions (3.3), each term satisfies (3.1) namely

3.6 $${\text{div}}_{x}\sigma^{i}=\rho \ddot{}u^{i},\;\sigma^{i}=\lambda \text{tr}(\varepsilon_{x}(u^{i}))1+2\mu \varepsilon_{x}(u^{i}),\;i\geq 0.$$

In contrast for the inner terms, the hierarchy of problems is obtained inserting the expansions (3.3) in (3.1) and using the differential operator $\nabla \to (e_{2}\frac{\partial \;}{\partial x_{2}}+\frac{1}{\eta }\nabla_{\;y})$ (according to the two scales $x_{2}$ and  y). Collecting the contributions in *η*^*i*^, we get the constitutive behaviours (**C**) and the equilibrium equations (**E**). At the zero and first orders, they read

3.7 $$\left(C)\;0=A\varepsilon_{\;y}(v^{0}),\;\tau^{0}=A(\varepsilon_{x_{2}}(v^{0})+\varepsilon_{\;y}(v^{1})\right)$$

and

3.8 $$(E)\;{\text{div}}_{\;y}\tau^{0}=0,\;{\text{div}}_{x_{2}}\tau^{0}+{\text{div}}_{\;y}\tau^{1}=\rho \ddot{}v^{0}.$$

For sake of simplicity, we have defined the following operators:

$$\varepsilon_{x_{1}}(u)=\left(\begin{array}{cc} \frac{\partial u_{1}}{\partial x_{1}} & \frac{1}{2}\frac{\partial u_{2}}{\partial x_{1}}\\ \frac{1}{2}\frac{\partial u_{2}}{\partial x_{1}} & 0 \end{array}\right),\;\varepsilon_{x_{2}}(u)=\left(\begin{array}{cc} 0 & \frac{1}{2}\frac{\partial u_{1}}{\partial x_{2}}\\ \frac{1}{2}\frac{\partial u_{1}}{\partial x_{2}} & \frac{\partial u_{2}}{\partial x_{2}} \end{array}\right),\;A\varepsilon =\lambda \text{tr}(\varepsilon )1+2\mu \varepsilon .$$

Eventually, the stress-free condition on the boundary of the void applies at each order

3.9 $$\tau^{0}n=\tau^{1}n=0\;\text{on}\;\;\partial V.$$

In the following, we shall solve (3.7)–(3.9) at the first two orders.

### (a) Zero-order resolution

Inverting the first relation of (**C**) in (3.7), we get that $\varepsilon_{\;y}(v^{0})=0$. Hence **v**^0^ is associated to a rigid body motion which is further reduced to a translation due to the periodicity of **v**^0^ with respect to $y_{2}$. Accordingly, we denote

$$v^{0}=V^{0}(x_{2},t).$$

In virtue of the matching conditions for **v**^0^ in (3.4), we deduce that

3.10 $$v_{k}^{0}=u_{k}^{0}(0^{+},x_{2},t)=u_{k}^{0}(0^{-},x_{2},t),\;k=1,2.$$

Now integrating ${\text{div}}_{\;y}\tau^{0}=0$, from (**E**) in (3.8), over $Y^{\text{m}}$ we get

$$0=\int_{Y^{\text{m}}}\text{d}\;y\;{\text{div}}_{\;y}\tau^{0}=\int_{y_{1}^{\text{m}}}\text{d}y_{2}\;\tau^{0}(y_{1}^{\text{m}},y_{2},t)\;e_{1}-\int_{-y_{1}^{\text{m}}}\text{d}y_{2}\;\tau^{0}(-y_{1}^{\text{m}},y_{2},t)\;e_{1},$$

where we have used the periodicity of **τ**^0^ with respect to $y_{2}$ and the stress-free condition $\tau^{0}n_{|\partial V}=0$ from (3.9). Passing to the limit $y_{1}^{\text{m}}\to +\infty$ and making use of the matching condition in (3.4), we eventually get

3.11 $$\sigma_{1k}^{0}(0^{+},x_{2},t)=\sigma_{1k}^{0}(0^{-},x_{2},t),\;k=1,2.$$

It is worth noting that, from (3.10) and (3.11), $\nabla_{x}u^{0}$ is continuous at $x_{1}=0$ (in the following we denote $\nabla_{x}u^{0}(0,x_{2},t)$ the limit). At the dominant order, the array of voids is invisible and we have to go to the first order to capture its effect.

### (b) First-order resolution

At this order, we have to solve a problem set on (**τ**^0^, **v**^1^) with respect to the spatial variable  y. From (3.7)–(3.8) along with the matching conditions on **τ**^0^ in (3.4), this problem reads

3.12 $$\begin{array}{rl} & {\text{div}}_{\;y}\tau^{0}=0,\;\tau^{0}=A\left(\varepsilon_{x_{2}}(u^{0}(0,x_{2},t))+\varepsilon_{\;y}(v^{1})\right),\;\text{in}Y\\ \;\text{and}\; & (v^{1},\tau^{0})\;y_{2}\text{-periodic},\;\tau^{0}n=0\;\text{on}\;\partial V,\;\lim_{y_{1}\to \pm \infty }\varepsilon_{\;y}(v^{1})=\varepsilon_{x_{1}}\left(u^{0}(0,x_{2},t)\right). \end{array}\}$$

In the definition of **τ**^0^ in (3.7), we have used that $v^{0}=u^{0}(0,x_{2},t)$, from (3.10). Next the limits of $\varepsilon_{\;y}(v^{1})$ for $y_{1}\to \pm \infty$ follow from (3.4) and using $\sigma^{0}=A(\varepsilon_{x_{1}}(u^{0})+\varepsilon_{x_{2}}(u^{0}))$. The problem (3.12) appears to be linear with respect to the two macroscopic loadings $\left(\varepsilon_{x_{1}}(u^{0}),\varepsilon_{x_{2}}(u^{0})\right)$. Hence the solution can be expressed as follow

3.13 $$v^{1}(x_{2},\;y,t)=y_{1}\frac{\partial u^{0}}{\partial x_{1}}(0,x_{2},t)+\sum_{i,j}\frac{\partial u_{i}^{0}}{\partial x_{j}}(0,x_{2},t)Q^{ij}(\;y)+V^{1}(x_{2},t),$$

where (*i*, *j*) ∈ {1, 2}^2^ and where the elementary vectors $Q^{ij}$ and associated tensors $T^{ij}$ satisfy the *elementary problems*

3.14 $$\begin{array}{rl} & {\text{div}}_{\;y}T^{ij}=0,\;T^{ij}=A(\varepsilon_{\;y}(Q^{ij})+e_{i}{⊗}^{s}e_{j}),\;\text{in}Y\\ \text{and}\; & (Q^{ij},T^{ij})\;y_{2}\text{-periodic},\;T^{ij}n=0\;\text{on}\;\partial V,\;\lim_{y_{1}\to \pm \infty }\varepsilon_{\;y}(Q^{ij})=0, \end{array}\}$$

where $e_{i}{⊗}^{s}e_{j}=1/2(e_{i}⊗e_{j}+e_{j}⊗e_{i})$. From (3.14), we see that up to a constant, $Q^{\text{1}2}=Q^{\text{2}1}$, leaving us with three elementary problems. The $Q^{ij}$ are defined up to constant vectors which can be fixed by imposing antisymmetric conditions at infinity, i.e.

3.15 $$\lim_{y_{1}\to \pm \infty }Q^{ij}=\pm \frac{B^{ij}}{2},$$

where the constant vectors $B^{ij}$ are *effective parameters* independent of the macroscopic loading. Now, using the matching conditions (3.5) for the displacement **u**^1^, it is easy to see from (3.13) that

3.16 $$\begin{array}{rl} u^{1}(0^{\pm },x_{2},t) & =\lim_{y_{1}\to \pm \infty }\left(v^{1}(x_{2},\;y,t)-y_{1}\frac{\partial u^{0}}{\partial x_{1}}(0,x_{2},t)\right)\\ & =\pm \frac{1}{2}\sum_{i,j}\frac{\partial u_{i}^{0}}{\partial x_{j}}(0,x_{2},t)B^{ij}+V^{1}(x_{2},t), \end{array}$$

from which we deduce that

3.17 $$u_{k}^{1}(0^{+},x_{2},t)-u_{k}^{1}(0^{-},x_{2},t)=\sum_{i,j}B_{k}^{ij}\frac{\partial u_{i}^{0}}{\partial x_{j}}(0,x_{2},t),$$

with (*i*, *j*, *k*) ∈ {1, 2}^3^. Yet we still have to derive the first-order condition for the normal stress. We start by expressing **τ**^0^ as a function of the elementary solutions $Q^{ij}$. From (3.12)–(3.13), and owing to $\varepsilon_{\;y}(v^{1})=\varepsilon_{x_{1}}(u^{0})(0,x_{2},t)+\sum_{i,j}\frac{\partial u_{i}^{0}}{\partial x_{j}}(0,x_{2},t)\varepsilon_{\;y}(Q^{ij})$, we get

$$\tau^{0}(x_{2},\;y,t)=\sigma^{0}(0,x_{2},t)+\sum_{i,j}\frac{\partial u_{i}^{0}}{\partial x_{j}}(0,x_{2},t)\;A\;\varepsilon_{\;y}(Q^{ij}(\;y)).$$

We use the above expression in the second equation of (**E**), (3.8), along with $v^{0}=u^{0}(0,x_{2},t)$ from (3.10). We also use from (3.6) that $0={\text{div}}_{x_{1}}\sigma^{0}+{\text{div}}_{x_{2}}\sigma^{0}-\rho \ddot{}u^{0}$. Doing so, we obtain

3.18 $${\text{div}}_{\;y}\tau^{1}={\text{div}}_{x_{1}}\sigma^{0}(0,x_{2},t)-\sum_{i,j}\frac{\partial^{2}u_{i}^{0}}{\partial x_{2}\partial x_{j}}(0,x_{2},t)\;A\;\varepsilon_{\;y}(Q^{ij}(\;y))e_{2}.$$

Now, integrating (3.18) over the cell Y and accounting for the matchings on **τ**^1^ in (3.5) leads to

$$0=(\sigma^{1}(0^{+},x_{2},t)-\sigma^{1}(0^{-},x_{2},t))e_{1}+e\varphi \;{\text{div}}_{x_{1}}\sigma^{0}(0,x_{2},t)+\sum_{i,j}C^{ij}\frac{\partial^{2}u_{i}^{0}}{\partial x_{2}\partial x_{j}}(0,x_{2},t),$$

where the vectors $C^{ij}$ are defined by

3.19 $$C^{ij}=\int_{Y}\text{d}\;y\;A\varepsilon_{\;y}(Q^{ij})\;e_{2},$$

and eventually, for *k* = 1, 2,

3.20 $$\sigma_{1k}^{1}(0^{+},x_{2},t)-\sigma_{1k}^{1}(0^{-},x_{2},t)=-e\varphi \;\frac{\partial \sigma_{1k}^{0}}{\partial x_{1}}(0,x_{2},t)-\sum_{i,j}C_{k}^{ij}\;\frac{\partial^{2}u_{i}^{0}}{\partial x_{2}\partial x_{j}}(0,x_{2},t).$$

### (c) Final up-to-first order conditions across enlarged interface

Following [20,21], up-to-first order conditions are constructed by recombining the zero and first-order conditions on (**u**^0^ + *η***u**^1^) from (3.10) and (3.11) and on (**σ**^0^ + *η***σ**^1^) from (3.17) and (3.20). It is worth noticing that these conditions implicitly involve the length *h* through *η* = *kh*. As the array thickness *e* = *O*(*h*) is of the same order of magnitude, it makes sense to account for its contribution too. Introduced in [23], *enlarged formulations* of the jump conditions has been shown to be stable, see also [24]. They are obtained by Taylor expansions of $u^{0}(0^{+},x_{2},t)$ and $\sigma^{0}(0^{+},x_{2},t)$. With the jump and mean defined in (2.3), we get

$$\begin{array}{rl} & [[u^{0}]]=e\bar{\frac{\partial u^{0}}{\partial x_{1}}}+O(\eta^{2}),\;\left[[u^{1}]]=\sum_{i,j}B^{ij}\bar{\frac{\partial u_{i}^{0}}{\partial x_{j}}}+O(\eta \right)\\ \;\text{and}\; & [[\sigma^{0}e_{1}]]=e\;\bar{\frac{\partial \sigma^{0}}{\partial x_{1}}}e_{1}+O(\eta^{2}),\;[[\sigma^{1}e_{1}]]=-e\varphi \;\bar{\frac{\partial \sigma^{0}}{\partial x_{1}}}e_{1}-\sum_{i,j}C^{ij}\bar{\frac{\partial u_{i}^{0}}{\partial x_{j}}}+O(\eta ), \end{array}$$

with the notations given by in (2.3). Combining the above equations and coming back to the real coordinate and time (***x***, *t*) leads to the final conditions in (2.2) which apply to the unique displacement $u^{\eta }=u^{0}+\eta u^{1}+O(\eta^{2})$ and stress $\sigma^{\eta }=\sigma^{0}+\eta \sigma^{1}+O(\eta^{2})$ defined in $x\in \{(x_{1},x_{2})\in R\setminus (0,e)\times R\}$.

### (d) Properties of the effective coefficients

**Lemma 3.1.**

*The effective parameters satisfy*

3.21 $$\begin{array}{rl} & B_{k}^{ij}=B_{k}^{ji},\;C_{k}^{ij}=C_{k}^{ji}, \end{array}$$

3.22 $$\begin{array}{rl} & C_{1}^{11}=C_{1}^{22}=0,\;C_{1}^{12}=\mu \varphi e \end{array}$$

3.23 $$\begin{array}{rl} \text{and}\; & \mu B_{2}^{11}=(\lambda +2\mu )B_{1}^{12},\;\lambda B_{1}^{11}-C_{2}^{11}=(\lambda +2\mu )B_{1}^{22}-\lambda \varphi e,\;\lambda B_{1}^{12}-C_{2}^{12}=\mu B_{2}^{22}. \end{array}$$

**Proof.**

The symmetries in (3.21) are straightforward since $Q^{ij}=Q^{ji}$ from (3.14), To establish (3.22), we integrate over Y the equilibrium equation ${\text{div}}_{\;y}T^{ij}=0$ in (3.14) after multiplication by $y_{2}e_{1}$ which is periodic along $y_{2}$. Integrating by part $\int_{Y^{\text{m}}}\text{d}\;y\;y_{2}e_{1}\cdot {\text{div}}_{\;y}T^{ij}=0$ and accounting for the periodicity of $T^{ij}$, the nullity of $T^{ij}n$ on ∂V and the limits $\lim_{y_{1}\to \pm \infty }T^{ij}=Ae_{i}{⊗}^{s}e_{j}$ result in

$$C_{\text{1}}^{ij}=(e_{1}⊗e_{2})\cdot A(e_{i}{⊗}^{s}e_{j})\varphi e=(\delta_{i1}\delta_{j2}+\delta_{i2}\delta_{j1})\mu \varphi e,$$

which provides the values of $C_{\text{1}}^{ij}$ in (3.22).

Next, to show (3.23), we need to introduce $D^{ij}=\int_{Y}\text{d}\;y\;A\varepsilon_{\;y}(Q^{ij})$. Repeating the procedure after multiplication by $y_{1}e_{1}$ provides the relations

3.24 $$D_{\text{1}1}^{ij}=(e_{1}⊗e_{1})\cdot A(e_{i}{⊗}^{s}e_{j})\varphi e=(\lambda \delta_{ik}\delta_{jk}+2\mu \delta_{i1}\delta_{j1})\varphi ,\Rightarrow D_{\text{1}1}^{\text{1}1}=(\lambda +\mu )\varphi e,\;D_{\text{1}1}^{\text{1}2}=0,\;D_{\text{1}1}^{\text{2}2}=\lambda \varphi e.$$

We now integrate ${\text{div}}_{\;y}T^{ij}=0$ after multiplication by $Q^{kl}$, specifically

$$0=\int_{Y}\text{d}\;y\;Q^{kl}\cdot {\text{div}}_{\;y}T^{ij}=-C_{kl}^{ij}-\int_{Y}\text{d}\;y\;\varepsilon_{\;y}(Q^{kl})\cdot A\varepsilon_{\;y}(Q^{ij})+B^{kl}\cdot A(e_{i}{⊗}^{s}e_{j})e_{1},$$

and we know that $A(e_{i}{⊗}^{s}e_{j})e_{1}=\lambda \text{tr}(e_{i}{⊗}^{s}e_{j})e_{1}+\mu (\delta_{1i}e_{j}+\delta_{1j}e_{i})$.

Introducing $q_{ijkl}=\int_{Y}\text{d}\;y\;\varepsilon_{\;y}(Q^{kl})\cdot A\varepsilon_{\;y}(Q^{ij})=\int_{Y}\text{d}\;y\;A\varepsilon_{\;y}(Q^{ij})\cdot \varepsilon_{\;y}(Q^{kl})$ we deduce that

3.25 $$q_{ijkl}=\lambda \delta_{ij}B_{\text{1}}^{kl}+\mu (\delta_{1i}B_{j}^{kl}+\delta_{1j}B_{i}^{kl})-D_{ij}^{kl}=\lambda \delta_{kl}B_{\text{1}}^{ij}+\mu (\delta_{1k}B_{l}^{ij}+\delta_{1l}B_{k}^{ij})-D_{kl}^{ij}.$$

Eventually, considering *q*_1112_, *q*_1122_ and *q*_1222_, it is easy to check that

$$\mu B_{\text{2}}^{\text{1}1}-D_{\text{1}2}^{\text{1}1}=(\lambda +2\mu )B_{\text{1}}^{\text{1}2}-D_{\text{1}1}^{\text{1}2},\;\lambda B_{\text{1}}^{\text{1}1}-D_{\text{2}2}^{\text{1}1}=(\lambda +2\mu )B_{\text{1}}^{\text{2}2}-D_{\text{1}1}^{\text{2}2},\;\lambda B_{\text{1}}^{\text{1}2}-D_{\text{2}2}^{\text{1}2}=\mu B_{\text{2}}^{\text{2}2}-D_{\text{1}2}^{\text{2}2},$$

which accounting for (3.24) and $C_{k}^{ij}=D_{\text{2}k}^{ij}$ provides (3.23). ▪

**Lemma 3.2.**

*For defects with a symmetry with respect to y*_1_ *or y*_2_, *we have*

3.26 $$B_{\text{2}}^{\text{1}1}=B_{\text{2}}^{\text{2}2}=B_{\text{1}}^{\text{1}2}=0,\;C_{\text{2}}^{\text{1}2}=0.$$

**Proof.**

Suppose first that the defects are symmetric with respect to $y_{2}$ (invariance $y_{2}\to -y_{2}$). The elementary problem on $Q^{\text{2}2}$ is associated with a loading $e_{2}⊗e_{2}=\varepsilon_{\;y}(y_{2}e_{2})$ and $y_{2}e_{2}$ is odd with respect to $y_{2}$. As a result the component $Q_{\text{2}}^{\text{2}2}$ in the same direction is also odd which further implies that its limit $B_{\text{2}}^{\text{2}2}=0$. For the same reasons, the elementary problem on $Q^{\text{1}2}$, associated with a loading $e_{1}{⊗}^{s}e_{2}=\varepsilon_{\;y}(y_{2}e_{1})$ (and $y_{2}e_{1}$ is odd with respect to $y_{2}$), produces an odd $Q_{\text{1}}^{\text{1}2}$, whence $B_{\text{1}}^{\text{1}2}=0$. Suppose now that the defects are symmetric with respect to $y_{1}$ (invariance $y_{1}\to -y_{1}$). The loading $y_{2}e_{2}$ associated with the problem on $Q^{\text{2}2}$ is even with respect to $y_{1}$, hence $Q_{\text{2}}^{\text{2}2}$ is also even. Since $\lim_{y_{1}\to \pm \infty }Q_{\text{2}}^{\text{2}2}=\pm B_{\text{2}}^{\text{2}2}/2$, we deduce that $B_{\text{2}}^{\text{2}2}=0$. Similarly, $Q_{\text{1}}^{\text{1}2}$ is an even function of *y*_1_ being associated with a loading $y_{2}e_{1}$. Therefore we get $B_{\text{1}}^{\text{1}2}=0$. Now, since we have in both cases $B_{\text{1}}^{\text{1}2}=B_{\text{2}}^{\text{2}2}=0$, using (3.23) we find that $B_{\text{2}}^{\text{1}1}=C_{\text{2}}^{\text{1}2}=0$. ▪

### (e) Remarks on the spring models

The jump conditions (2.2), along with (3.22) and (3.23), are more general than the spring models except in particular or limiting cases which are listed below.

— *Elastic waves at normal incidence.* In this case, ∂/∂*x*_2_ = 0 resulting in
  3.27 $$\begin{array}{rl} & [[u_{1}]]=h\frac{e+B_{\text{1}}^{\text{1}1}}{\lambda +2\mu }\;\bar{\sigma_{11}}+h\frac{B_{\text{1}}^{\text{1}2}}{\mu }\;\bar{\sigma_{12}},\;[[\sigma_{11}]]=-\rho \omega^{2}he(1-\varphi )\bar{u_{1}}\\ \;\text{and}\; & [[u_{2}]]=h\frac{B_{\text{2}}^{\text{1}1}}{\lambda +2\mu }\bar{\sigma_{11}}+h\frac{e+B_{\text{2}}^{\text{1}2}}{\mu }\;\bar{\sigma_{12}},\;[[\sigma_{12}]]=-\rho \omega^{2}he(1-\varphi )\bar{u_{2}}. \end{array}\}$$
  We find the *enlarged version* of the inertial terms introduced by Baik & Thomson [3] in the jumps of the normal stresses. The jumps of the displacements involve four compliances from which the four stiffnesses can be deduced by inversion. This *mass-spring model* is as accurate as (2.2) but restricted to normal incidence.
— *Small thickness e≪h(e≪1).* Noticing that the $B_{\text{1}}^{\text{2}2}$ and $B_{\text{2}}^{\text{1}2}$ in (3.15), being the elastic analogues of the *blockage coefficients* in acoustic, do not vanish for vanishing *e*, noticing on the contrary that the $C_{k}^{ij}$ in (3.19) and ($B_{\text{2}}^{\text{1}1}$, $B_{\text{2}}^{\text{2}2},B_{\text{1}}^{\text{1}2}$) from (3.26) vanish for vanishing *e* (vanishing *e* produces symmetry with respect to $y_{2}$), and neglecting the terms *O*(*e*) makes that (2.2) simplifies to
  3.28 $$\begin{array}{rl} & \sigma_{11}(0,x_{2})=\kappa_{1}[[u_{1}]],\;\sigma_{12}(0,x_{2})=\kappa_{2}[[u_{2}]]\\ \;\text{and}\; & \kappa_{1}=\frac{\lambda +2\mu }{hB_{\text{1}}^{\text{1}1}},\;\kappa_{2}=\frac{\mu }{hB_{\text{2}}^{\text{1}2}}, \end{array}\}$$
  with normal stresses continuous at *x*_1_ = 0. (We have used that (3.23) gives $\lambda B_{\text{1}}^{\text{1}1}=(\lambda +2\mu )B_{\text{1}}^{\text{2}2}$ up to O(e).) The above *massless spring model* is an approximation of our model up to O(e). It involves two, normal and tangential, stiffnesses which differ as $B_{\text{1}}^{\text{1}1}/(\lambda +2\mu )$ and $B_{\text{2}}^{\text{1}2}/\mu$ do; these later still depend on e as they depend although weakly to the geometry of the thin voids.
— *Aligned cracks (e=0).* This case is the limit of the previous one. We have checked that for vanishing e, we have *κ*_1_ = *κ*_2_ = *κ* with
  3.29 $$\kappa =\frac{\mu }{h}\frac{\lambda +\mu }{\lambda +2\mu }\frac{\pi }{\log{\left(\cos(\pi \varphi /2)\right)}^{-1}},$$
  and we recover the model of Angel & Achenbach [11] with a single, explicit, stiffness.

## 4. Energetic properties of the effective model

We come back to the general case of an imperfect interface ruled by the jump conditions (2.2) within a domain Ω. To derive the balance of energy in the effective problem, we multiply the equilibrium equation (first equation in (2.1)) by $\dot{u}$ and the constitutive behaviour (second equation in (2.1)) by $\varepsilon_{x}(\dot{u})$, and we sum up. Doing so and after an integration by part, we get

4.1 $$\begin{array}{cc} \; & \frac{d}{dt}E_{\Omega }(t)+\Phi_{\partial \Omega }(t)=0,\;\\ \; & \;\;E_{\Omega }(t)=\frac{1}{2}\int_{\Omega }dx\;\left(\rho \dot{u}\cdot \dot{u}+\lambda {\left(\mathrm{tr}(\varepsilon_{x}(u))\right)}^{2}+2\mu \varepsilon_{x}(u)\cdot \varepsilon_{x}(u)\right)\;\\ \mathrm{and}\; & \Phi_{\partial \Omega }(t)=-\int_{\partial \Omega }ds\;\sigma \dot{u}\cdot n.\; \end{array}\}$$

$E_{\Omega }$ is the classical elastic energy and Φ_∂Ω_ is the flux of the elastic Poynting vector $\left(\sigma \dot{u}\right)$. Usually, the fields $\dot{u}$ and **σn** are continuous or they vanish on ∂Ω. Here, the imperfect interface produces a discontinuity of these fields, hence it makes a contribution Φ_Γ_ to Φ_∂Ω_ which reads

4.2 $$\Phi_{\Gamma }(t)=\int \text{d}x_{2}\;[[\dot{u}\cdot \sigma e_{1}]].$$

**Lemma 4.1.**

*There exists* $E_{\Gamma }(t)\geq 0$ *termed interfacial energy such that* $\Phi_{\Gamma }(t)=(\text{d}/\text{d}t)E_{\Gamma }(t)$ *and defined by*

4.3  EΓ(t)=EK(t)+EP(t)with EK(t)=h2∫Γdx2 e(1−φ)ρu¯˙⋅u¯˙, EP(t)=h2∫Γdx2 [E11 ε11¯2+E12 ε12¯2+E22 ε22¯2+E1112 ε11¯ ε12¯ +E1122 ε11¯ ε22¯+E1222 ε12¯ ε22¯],}

$\left(E_{K},E_{P}\right)$ *being the kinetic and potential energies associated with the interface, with*

4.4 $$\begin{array}{rl} & E_{11}=(\lambda +2\mu )(e+B_{1}^{11}),\;E_{12}=4\mu (e+B_{2}^{12}),\;E_{22}=(\lambda +2\mu )e(1-\varphi )+\lambda B_{1}^{22}+C_{2}^{22}\\ \text{and} & \;E_{1112}=4(\lambda +2\mu )B_{1}^{12},\;\;E_{1122}=2\lambda (e+B_{1}^{11}),\;E_{1222}=4\lambda B_{1}^{12}. \end{array}\}$$

*Besides*, $\left(E_{K},E_{P}\right)$ *satisfies*

4.5 $$E_{K}\geq 0,\;E_{P}\geq \frac{e}{1-\varphi }\left(\frac{{\bar{\sigma_{11}}}^{2}}{\lambda +2\mu }+\frac{{\bar{\sigma_{12}}}^{2}}{\mu }\right)\geq 0.$$

**Proof.**

We use (2.2) in $\Phi_{\Gamma }=\int_{\Gamma }\text{d}x_{2}\;([[\dot{u_{k}}]]\bar{\sigma_{1k}}+\dot{\bar{u_{k}}}[[\sigma_{1k}]])$, hence

4.6 $$\Phi_{\Gamma }(t)=h\int_{\Gamma }\text{d}x_{2}\;\left[-e(1-\varphi )\underset{\text{(IP)}}{\underbrace{\frac{\partial \bar{\sigma_{2k}}}{\partial x_{2}}\;\dot{\bar{u_{k}}}}}+e(1-\varphi )\rho {\dot{\bar{u}}}_{k}{\ddot{\bar{u}}}_{k}-C_{k}^{ij}\underset{\text{(IP)}}{\underbrace{\frac{\partial \bar{\varepsilon_{ij}}}{\partial x_{2}}\;\dot{\bar{u_{k}}}}}+e\;\frac{\partial {\dot{\bar{u}}}_{k}}{\partial x_{1}}\;\bar{\sigma_{1k}}+B_{k}^{ij}\;\dot{\bar{\varepsilon_{ij}}}\;\bar{\sigma_{1k}}\right].$$

(We have used that $\bar{\partial \sigma_{1k}}/\partial x_{1}=-(\partial \bar{\sigma_{2k}}/\partial x_{2})+\rho {\ddot{\bar{u}}}_{k}$.) Integrating by parts the two terms (IP) in (4.6) provides $\Phi_{\Gamma }(t)=h\int_{\Gamma }\text{d}x_{2}\;[2e(1-\varphi )\rho \dot{\bar{u_{k}}}\;\ddot{\bar{u_{k}}}+T_{\text{1}}(x_{2},t)+T_{\text{2}}(x_{2},t)]$ with $T_{\text{1}}(x_{2},t)=C_{k}^{ij}\;\bar{\varepsilon_{ij}}(\partial \dot{\bar{u_{k}}}/\partial x_{2})+B_{k}^{ij}\dot{\bar{\varepsilon_{ij}}}\;\bar{\sigma_{1k}}$ and $T_{\text{2}}(x_{2},t)=e(1-\varphi )\bar{\sigma_{2k}}\;(\partial \dot{\bar{u_{k}}}/\partial x_{2})+(e\partial \dot{\bar{u_{k}}}/\partial x_{1})\;\bar{\sigma_{1k}}$. Using further (3.22)–(3.23), *T*_1_ reads

$$\begin{array}{c} T_{1}(x_{2},t)=\frac{1}{2}\frac{\partial }{\partial t}[B_{1}^{11}\left(2\lambda \bar{\varepsilon_{22}}+(\lambda +2\mu )\bar{\varepsilon_{11}}\right)\bar{\varepsilon_{11}}+4B_{1}^{12}\left(\lambda \bar{\varepsilon_{22}}+(\lambda +2\mu )\bar{\varepsilon_{11}}\right)\bar{\varepsilon_{12}}\;\\ \;\;\;\;+4\mu B_{2}^{12}{\bar{\varepsilon_{12}}}^{2}+(\lambda B_{1}^{22}+C_{2}^{22}){\bar{\varepsilon_{22}}}^{2}]+e\varphi \left[\lambda \bar{\varepsilon_{11}}\;\dot{\bar{\varepsilon_{22}}}+2\mu \bar{\varepsilon_{12}}\;\frac{\partial \dot{\bar{u_{1}}}}{\partial x_{2}}\right],\; \end{array}$$

and using the form of **σ** in (2.1), we also have

$$T_{\text{2}}(x_{2},t)=\frac{e}{2}\;\frac{\partial \;}{\partial t}[2\lambda \bar{\varepsilon_{11}}\;\bar{\varepsilon_{22}}+(1-\varphi )(\lambda +2\mu ){\bar{\varepsilon_{22}}}^{2}+4\mu {\bar{\varepsilon_{12}}}^{2}+(\lambda +2\mu ){\bar{\varepsilon_{11}}}^{2}]-e\varphi \left[\lambda \bar{\varepsilon_{11}}\;\dot{\bar{\varepsilon_{22}}}+2\mu \bar{\varepsilon_{12}}\;\frac{\partial \dot{\bar{u_{1}}}}{\partial x_{2}}\right],$$

hence the result (4.3).

The proof of the positiveness of $E_{P}$ by means of (4.5) is postponed to appendix A. ▪

The positivity of $E_{\Gamma }(t)$ guarantees the stability of the effective problem, which basically means that ***u*** and **σ** are bounded [23]. In practice, $E_{\Gamma }>0$ prevents from numerical instabilities which happen when an increase in time of $E_{\text{Ω}}$ (*t*) → +∞ is compensated by $E_{\Gamma }\to -\infty$, see an illustration in [25].

## 5. A scattering problem, validation and discussion

In this section, we consider the harmonic regime with time dependence e^−i*ωt*^; we shall inspect the validity of our effective model by means of comparison with direct numerics based on a multimodal method for two-dimensional elasticity [22]. We restrict ourselves to rectangular voids, whose symmetry makes that, from (3.22), (3.23) and (3.26), the jump conditions (2.2) simplify to

5.1 $$\begin{array}{rl} & [[u_{1}]]=h(e+B_{\text{1}}^{\text{1}1})\;\bar{\frac{\partial u_{1}}{\partial x_{1}}}+hB_{\text{1}}^{\text{2}2}\;\frac{\partial \bar{u_{2}}}{\partial x_{2}},\;[[\sigma_{11}]]=he(1-\varphi )\bar{\frac{\partial \sigma_{11}}{\partial x_{1}}}-h\mu \varphi e\frac{\partial \;}{\partial x_{2}}\left(\frac{\partial \bar{u_{1}}}{\partial x_{2}}+\bar{\frac{\partial u_{2}}{\partial x_{1}}}\right)\\ \;\text{and}\; & [[u_{2}]]=h(e+B_{\text{2}}^{\text{1}2})\;\bar{\frac{\partial u_{2}}{\partial x_{1}}}+hB_{\text{2}}^{\text{1}2}\;\frac{\partial \bar{u_{1}}}{\partial x_{2}},\;[[\sigma_{12}]]=he(1-\varphi )\bar{\frac{\partial \sigma_{12}}{\partial x_{1}}}-h\frac{\partial \;}{\partial x_{2}}\left(C_{\text{2}}^{\text{1}1}\bar{\frac{\partial u_{1}}{\partial x_{1}}}+C_{\text{2}}^{\text{2}2}\frac{\partial \bar{u_{2}}}{\partial x_{2}}\right). \end{array}\}$$

We have in addition from (3.23) that $\lambda B_{\text{1}}^{\text{1}1}-C_{\text{2}}^{\text{1}1}=(\lambda +2\mu )B_{\text{1}}^{\text{2}2}-\lambda \varphi e$.

### (a) Scattering coefficients and energy fluxes

In two dimensions, the wavefield is defined in terms of the elastic scalar potentials Φ and Ψ such that $u=\nabla \Phi +\nabla \times (\Psi e_{3})$. The incident wave in the matrix derives from the potentials

5.2 $$\begin{array}{rl} & \Phi^{\text{i}nc}(x)=A_{\text{L}}\;{\text{e}}^{\text{i}\alpha_{\text{L}}x_{1}}\;{\text{e}}^{\text{i}\beta x_{2}},\;\Psi^{\text{i}nc}(x)=A_{\text{T}}\;{\text{e}}^{\text{i}\alpha_{\text{T}}x_{1}}\;{\text{e}}^{\text{i}\beta x_{2}},\\ & \text{with }\;(\alpha_{\text{L}},\beta )=k_{\text{L}}\;(\cos\theta_{\text{L}},\sin\theta_{\text{L}}),\;(\alpha_{\text{T}},\beta )=k_{\text{T}}\;(\cos\theta_{\text{T}},\sin\theta_{\text{T}}), \end{array}\}$$

and $k_{\text{L}}=\sqrt{\rho /\lambda +2\mu }\;\omega$, $k_{\text{T}}=\sqrt{\rho /\mu }\;\omega$. The domain is unbounded and the radiation conditions are accounted for by imposing that the scattered field is outgoing; in the effective problem, the solution simply reads

5.3 $$\begin{array}{rl} & \text{for }x_{1}<0,\;\Phi (x)=\Phi^{\text{i}nc}(x)+r_{\Phi }\;{\text{e}}^{-\text{i}\alpha_{\text{L}}x_{1}}\;{\text{e}}^{\text{i}\beta x_{2}},\;\Psi (x)=\Psi^{\text{i}nc}(x)+r_{\Psi }\;{\text{e}}^{-\text{i}\alpha_{\text{T}}x_{1}}\;{\text{e}}^{\text{i}\beta x_{2}},\\ & \text{for }x_{1}>e,\;\Phi (x)=t_{\Phi }\;{\text{e}}^{\text{i}\alpha_{\text{L}}(x_{1}-e)}\;{\text{e}}^{\text{i}\beta x_{2}},\;\Psi (x)=t_{\Psi }e^{\text{i}\alpha_{\text{T}}(x_{1}-e)}\;{\text{e}}^{\text{i}\beta x_{2}}, \end{array}\}$$

with

**Figure 3.**
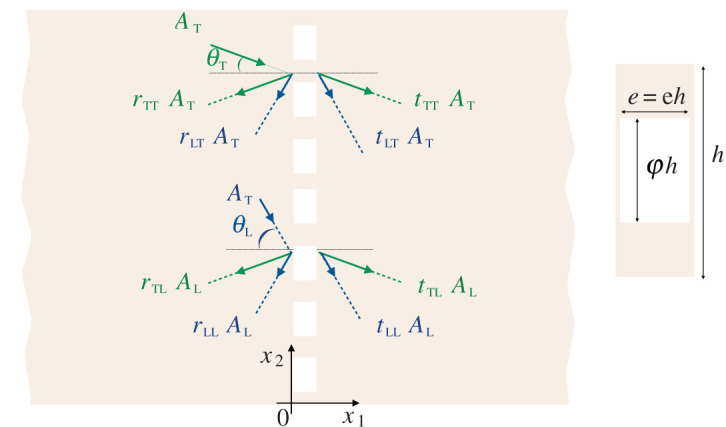
Scattering of an incident wave by an array of voids, with spacing *h*. The rectangular voids have a thickness e=eh (along *x*_1_) and width φ*h* (along *x*_2_). (Online version in colour.)

5.4 $$\left(\begin{array}{l} r_{\Phi }\\ r_{\Psi } \end{array}\right)=\left(\begin{array}{ll} r_{\text{L}L} & r_{\text{L}T}\\ r_{\text{T}L} & r_{\text{T}T} \end{array}\right)\left(\begin{array}{l} A_{\text{L}}\\ A_{\text{T}} \end{array}\right),\;\left(\begin{array}{l} t_{\Phi }\\ t_{\Psi } \end{array}\right)=\left(\begin{array}{ll} t_{\text{L}L} & t_{\text{L}T}\\ t_{\text{T}L} & t_{\text{T}T} \end{array}\right)\left(\begin{array}{l} A_{\text{L}}\\ A_{\text{T}} \end{array}\right),$$

involving the 4 scattering coefficients in reflexion and the 4 scattering coefficients in transmission (figure 3). The jumps in (5.1) provide four relations which can be used for *A*_T_ = 0, *A*_L_ = 1, then for *A*_T_ = 1, *A*_L_ = 0, resulting in the eight scattering coefficients. Their expressions, although explicit, are quite involved. They are given in the appendix 2, see (6)–(8). It is easy to see that the scattering coefficients ensure the reciprocity and less easy but straightforward to see they ensure the conservation of the fluxes. Conservation of the fluxes is measured in terms of the conservation of the reflected and transmitted fluxes of the Poynting vector defined in (4.1). Once written in the harmonic regime, it imposes

5.5 $$\begin{array}{rl} & ℜ\left[\alpha_{\text{T}}(|r_{\text{T}T}{|}^{2}+|t_{\text{T}T}{|}^{2})+\alpha_{\text{L}}(|r_{\text{L}T}{|}^{2}+|t_{\text{L}T}{|}^{2})\right]A_{\text{T}}^{2}=ℜ\left[\alpha_{\text{T}}A_{\text{T}}^{2}\right]\\ \;\text{and}\; & ℜ\left[\alpha_{\text{L}}(|r_{\text{L}L}{|}^{2}+|t_{\text{L}L}{|}^{2})+\alpha_{\text{T}}(|r_{\text{T}L}{|}^{2}+|t_{\text{T}L}{|}^{2})\right]A_{\text{L}}^{2}=ℜ\left[\alpha_{\text{L}}A_{\text{L}}^{2}\right], \end{array}\}$$

(where ℜ means real part) and the results holds by summing the two equations for an incident flux $ℜ\{\alpha_{\text{T}}A_{\text{T}}^{2}+\alpha_{\text{L}}A_{\text{L}}^{2}\}$. For an incident longitudinal wave (I=L) or an incident transverse wave (I=T), we define the normalized energy fluxes in reflection $\phi_{\text{O}I}^{r}$ and in transmission $\phi_{\text{O}I}^{t}$ with O=L,T by

5.6 $$\begin{array}{rl} & \Phi_{\text{T}T}^{r}=|r_{\text{T}T}{|}^{2},\Phi_{\text{T}T}^{t}=|t_{\text{T}T}{|}^{2},\Phi_{\text{L}T}^{r}=ℜ\left[\frac{\alpha_{\text{L}}}{\alpha_{\text{T}}}\right]|r_{\text{L}T}{|}^{2},\Phi_{\text{L}T}^{t}=ℜ\left[\frac{\alpha_{\text{L}}}{\alpha_{\text{T}}}\right]|t_{\text{T}L}{|}^{2},\\ \text{and}\; & \Phi_{\text{L}L}^{r}=|r_{\text{L}L}{|}^{2},\Phi_{\text{L}L}^{t}=|t_{\text{L}L}{|}^{2},\Phi_{\text{T}L}^{r}=ℜ\left[\frac{\alpha_{\text{T}}}{\alpha_{\text{L}}}\right]|r_{\text{T}L}{|}^{2},\Phi_{\text{T}L}^{t}=ℜ\left[\frac{\alpha_{\text{T}}}{\alpha_{\text{L}}}\right]|t_{\text{T}L}{|}^{2}, \end{array}\}$$

and the conservation of the fluxes is ensured by

5.7 $$\Phi_{\text{T}T}^{r}+\Phi_{\text{T}T}^{t}+\Phi_{\text{L}T}^{r}+\Phi_{\text{L}T}^{t}=1,\;\Phi_{\text{L}L}^{r}+\Phi_{\text{L}L}^{t}+\Phi_{\text{T}L}^{r}+\Phi_{\text{T}L}^{t}=1.$$

### (b) Results

The material and geometrical parameters are *ρ* = *μ* = *λ* = 1, *h* = 1 and we shall consider different (e,φ). We shall inspect the effectiveness of our model in the range *k*_T_ *h* ∈ (0, 3) hence well beyond the expected range of validity of homogenization (which assumes *η* = *k*_T_ *h* ≪ 1).

In the first exemple for square voids e=φ=0.5, we shall take the opportunity to illustrate the consequence of the *enlarged* formulation (5.1) in which the jumps and means are expressed for an interface with boundaries at *x*_1_ = 0 and *e*, (2.3). More usually, the *zero-thickness* formulation, that is provided directly by the asymptotic analysis, is used for an interface with boundaries at *x*_1_ = 0^−^ and 0^+^. (It involves jumps (*f*(0^+^, *x*_2_) − *f*(0^−^, *x*_2_)) and mean values 1/2(*f*(0^+^, *x*_1_) + *f*(0^−^, *x*_2_)).) This formulation does not account for the Taylor expansions used in §3c. Hence it is obtained by simply removing in (5.1) the contributions due to Taylor expansion, e.g. $he\bar{\partial_{x_{1}}u_{1}}$ in [[*u*_1_]].

Then, we shall move on the case of aligned thin voids, with e≪h whose limiting case are cracks, i.e. e=0. This allows us to inspect in addition the effectiveness of the *massless-spring models* (3.28) and (3.29).

For the different geometries considered in this section, the effective parameters entering in (5.1) are reported below.
5.8
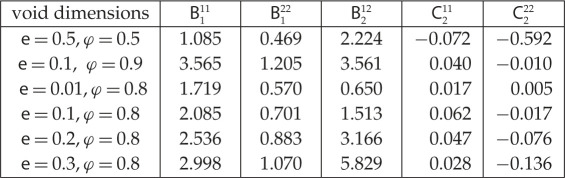

(They satisfy the relation $C_{\text{2}}^{\text{1}1}=\lambda B_{\text{1}}^{\text{1}1}-(\lambda +2\mu )B_{\text{1}}^{\text{2}2}+\lambda \varphi e$, (3.22).)

#### (i) Scattering by square voids

**Figure 4.**
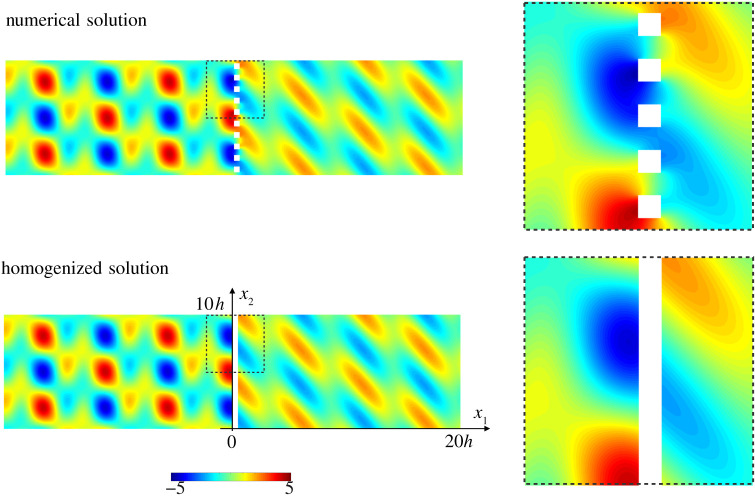
Scattering of a wave (with *A*_L_ = 3, *A*_T_ = 1) at incidence $\theta_{\text{T}}=30^{\circ }\text{--}\theta_{\text{L}}=60^{\circ }$ and *k*_T_ *h* = 2 by an array of square voids (e=φ=0.5) – Field of the displacement *u*_1_(*x*_1_, *x*_2_); the insets show zooms of the near field. (Online version in colour.)

**Figure 5.**
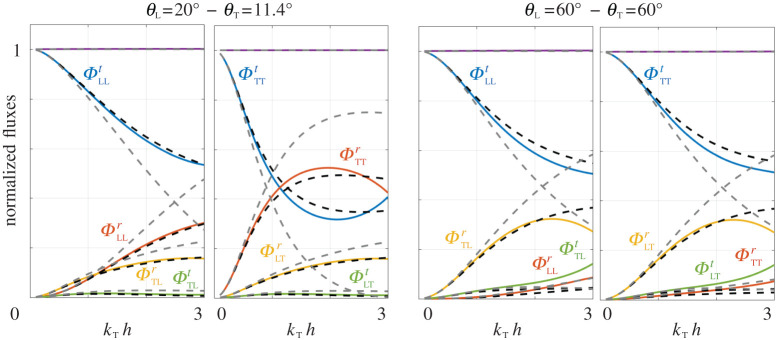
Normalized fluxes (5.6) against the non-dimensional frequency *k*_T_ *h* for an array of square voids with e=φ=0.5, at two incidences; plain lines from direct numerics and dashed black lines from our model (5.1) with *enlarged formulation*. For comparison, dashed grey lines show the result of the model in the *zero-thickness formulation*. (Purple curves show that the conservation of the fluxes (5.7) is satisfied in the numerics and in the effective problems.) (Online version in colour.)

Here we consider square voids with φ=e=0.5, hence an array with significant thickness. We start by reporting in figure 4 the displacement *u*_1_(*x*_1_, *x*_2_) for an incident wave of the form (5.2) with *k*_T_ *h* = 2, (*A*_L_ = 3, *A*_T_ = 1), (*θ*_T_ = 30^°^, *θ*_L_ = 60^°^) producing *k*_Lsin_*θ*_L_ = *k*_Tsin_*θ*_T_. The qualitative agreement between the field computed numerically and that given by (5.3) is excellent. In the actual problem, the near field is composed of many evanescent modes while in the homogenized problem, it is accounted for by the jump conditions. (Hence, in the close vicinity of the array, the variation of the evanescent field is not reproduced by construction, see insets of figure 4.) More quantitatively, we report in figure 5 the normalized fluxes against *k*_T_ *h* obtained numerically (plain lines) and from our model (black dashed lines). Our model, in its *enlarged formulation*, has an excellent accuracy up to *k*_T_ *h* ∼ 1 and a reasonably good one up to *k*_T_ *h* ∼ 2. By contrast, the validity of the model in the *zero-thickness formulation* (grey dashed lines) is severely limited to the low frequencies. It is a good news that our model which has good energetic properties has in addition a better effectiveness.

#### (ii) Scattering by thin voids: limiting cases of cracks

**Figure 6.**
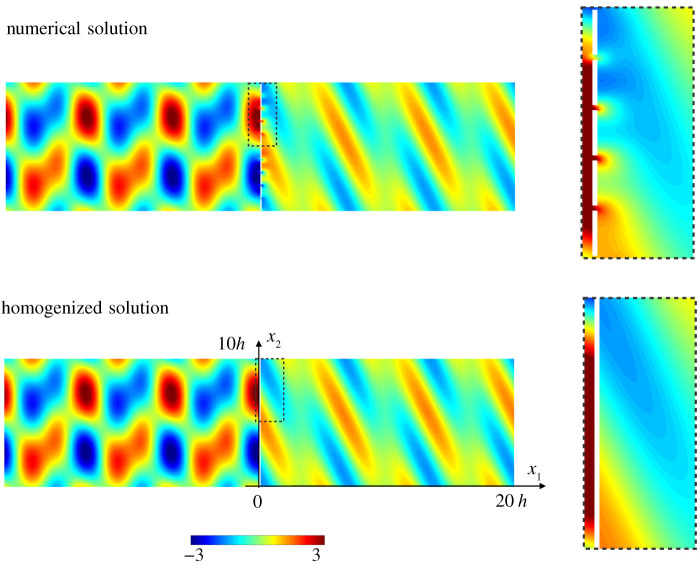
Scattering of a wave (*k*_T_ *h* = 2, *A*_L_ = 1, *A*_T_ = 1, $\theta_{\text{T}}=20^{\circ }\text{--}\theta_{\text{L}}=36.3^{\circ }$) by an array of thin voids (e=0.1, φ = 0.9)—same representation as in figure 3; the fields for *x*_1_ > 0 have been multiplied by a factor 2 for visibility. (Online version in colour.)

**Figure 7.**
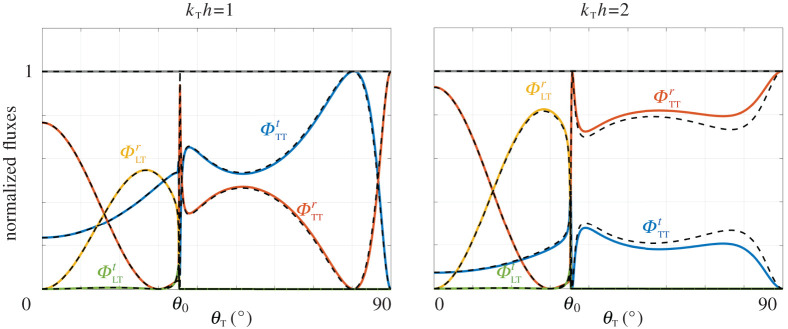
Normalized fluxes against the angle *θ*_T_ of an incident T-wave (e=0.1, φ = 0.9). Above the critical angle *θ*_0_ = 35.26^°^ of total reflection of the L-wave, the conversion of T- to L-wave is not possible. (Online version in colour.)

Thin voids are efficient scatterers if φ is large. We start with e=0.1 and φ = 0.9. As in the previous example, the displacement field computed numerically, and reported in figure 6, is nicely reproduced by our model despite the relatively large *k*_T_ *h* = 2 value. Again, this holds except in the close vicinity of the array and in the present case, the evanescent field is particularly strong due to the thinness of the connected parts between two voids. Also well reproduced are the variations of the fluxes against the angle *θ*_T_ of an incident T-wave (figure 7). This includes the sharp variations near the critical angle *θ*_0_ of total reflection of the L-wave ($\theta_{0}=\sin^{-1}\sqrt{\frac{\mu }{\lambda +2\mu }}≃35.26^{\circ }$). (For *θ*_T_ > *θ*_0_, L-waves are evanescent hence they contribute to the evanescent field but they do not transport energy. Accordingly, $\Phi_{\text{L}T}^{r}=\Phi_{\text{L}T}^{t}=0$ and the T-waves support alone the energy in reflection and in transmission with $\Phi_{\text{T}T}^{r}+\Phi_{\text{T}T}^{t}=1$.)

We now envision the simplified *massless-spring* models (3.28) and (3.29) when e≪1. The scattering coefficients have a simple form, see (9); defining $\xi =\sqrt{(\lambda +2\mu )/\mu }$ and

5.9 $$\begin{array}{rl} & \text{F}(k_{\text{L}}h)=\frac{2\xi^{2}}{k_{\text{L}}hB_{\text{1}}^{\text{1}1}},\;\text{G}(k_{\text{L}}h)=\frac{2\xi }{k_{\text{L}}hB_{\text{2}}^{\text{1}2}}\\ \;\text{and}\; & d_{\text{F}}=\sin2\theta_{\text{L}}\sin2\theta_{\text{T}}+\xi^{2}\cos^{2}2\theta_{\text{T}}+\text{i}\text{F}\cos\theta_{\text{L}},\\ & d_{\text{G}}=\sin2\theta_{\text{L}}\sin2\theta_{\text{T}}+\xi^{2}\cos^{2}2\theta_{\text{T}}+\text{i}\text{G}\cos\theta_{\text{T}}, \end{array}\}$$

the 8 scattering coefficients in (5.4) are given by

5.10 $$\begin{array}{rl} & r_{\text{L}L}=\frac{1}{2}\left[\sin2\theta_{\text{L}}\sin2\theta_{\text{T}}-\xi^{2}\cos^{2}2\theta_{\text{T}}\right]\left[\frac{1}{d_{\text{F}}}+\frac{1}{d_{\text{G}}}\right]+\frac{\text{i}}{2}\left[\frac{\text{F}\cos\theta_{\text{L}}}{d_{\text{F}}}-\frac{\text{G}\cos\theta_{\text{T}}}{d_{\text{G}}}\right],\\ & t_{\text{L}L}=\frac{1}{2}\left[\sin2\theta_{\text{L}}\sin2\theta_{\text{T}}-\xi^{2}\cos^{2}2\theta_{\text{T}}\right]\left[\frac{1}{d_{\text{F}}}-\frac{1}{d_{\text{G}}}\right]+\frac{\text{i}}{2}\left[\frac{\text{F}\cos\theta_{\text{L}}}{d_{\text{F}}}+\frac{\text{G}\cos\theta_{\text{T}}}{d_{\text{G}}}\right],\\ & r_{\text{T}T}=\frac{1}{2}\left[\sin2\theta_{\text{L}}\sin2\theta_{\text{T}}-\xi^{2}\cos^{2}2\theta_{\text{T}}\right]\left[\frac{1}{d_{\text{F}}}+\frac{1}{d_{\text{G}}}\right]-\frac{\text{i}}{2}\left[\frac{\text{F}\cos\theta_{\text{L}}}{d_{\text{F}}}-\frac{\text{G}\cos\theta_{\text{T}}}{d_{\text{G}}}\right],\\ & t_{\text{T}T}=-\frac{1}{2}\left[\sin2\theta_{\text{L}}\sin2\theta_{\text{T}}-\xi^{2}\cos^{2}2\theta_{\text{T}}\right]\left[\frac{1}{d_{\text{F}}}-\frac{1}{d_{\text{G}}}\right]+\frac{\text{i}}{2}\left[\frac{\text{F}\cos\theta_{\text{L}}}{d_{\text{F}}}+\frac{\text{G}\cos\theta_{\text{T}}}{d_{\text{G}}}\right],\\ & r_{\text{T}L}=\sin2\theta_{\text{L}}\cos2\theta_{\text{T}}\left[\frac{1}{d_{\text{F}}}+\frac{1}{d_{\text{G}}}\right],\;t_{\text{T}L}=-\sin2\theta_{\text{L}}\cos2\theta_{\text{T}}\left[\frac{1}{d_{\text{F}}}-\frac{1}{d_{\text{G}}}\right],\\ & r_{\text{L}T}=-\xi^{2}\sin2\theta_{\text{T}}\cos2\theta_{\text{T}}\left[\frac{1}{d_{\text{F}}}+\frac{1}{d_{\text{G}}}\right],\;t_{\text{L}T}=-\xi^{2}\sin2\theta_{\text{T}}\cos2\theta_{\text{T}}\left[\frac{1}{d_{\text{F}}}-\frac{1}{d_{\text{G}}}\right], \end{array}\}$$

see (9). For φ → 1, F and G vanish (since $B_{\text{1}}^{\text{1}1}$ and $B_{\text{2}}^{\text{1}2}$ diverge) and *d*_F_ = *d*_G_. This produces vanishing transmission coefficients and we recover the four reflection coefficients of a stress-free surface. In the other limit, for collapsing voids φ → 0, F and G diverge (since $B_{\text{1}}^{\text{1}1}=B_{\text{2}}^{\text{1}2}=0$) and Fcos*θ*_L_/*d*_F_ = Gcos*θ*_T_/*d*_G_ = 1/i; we recover a perfect transmission without L-T polarization conversion.

**Figure 8.**
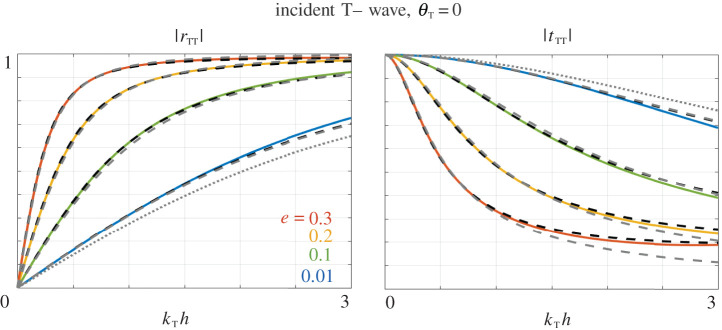
Scattering coefficients against non-dimensional frequency *k*_T_ *h* for thin voids φ = 0.8, for a T-wave at normal incidence (and *r*_LT_ = *t*_LT_ = 0). Plain lines show *r*_TT_ and *t*_TT_ computed numerically, dashed black lines from our model (5.1), dashed grey lines from the *massless-spring* model (3.28) and dotted grey line in the limit (3.29). (Online version in colour.)

**Figure 9.**
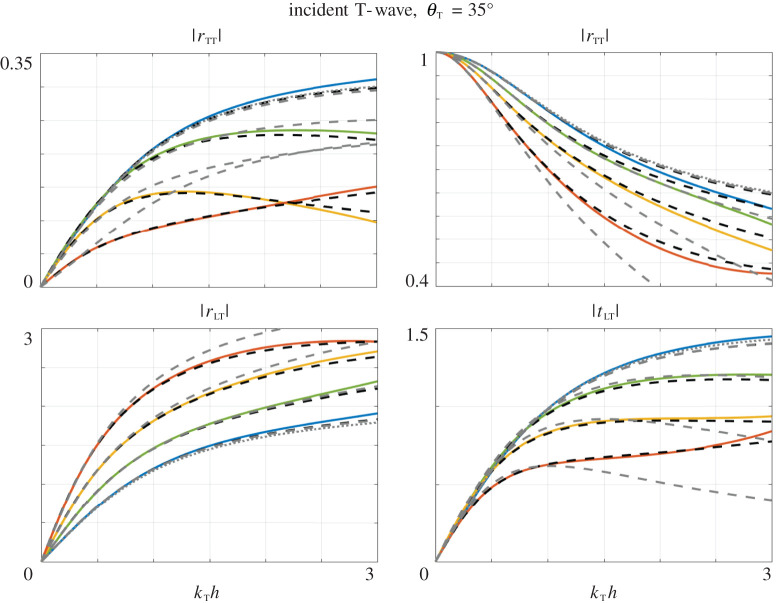
Same representation as in figure 8 for a T-wave at oblique incident. (Online version in colour.)

We have thus, in addition to our model (5.1), an approximate *massless-spring* formulation for e≪1, (3.28) involving two stiffnesses, and its limit e=0 resulting in a single, explicit, stiffness (3.29). Many examples of the variations of the scattering coefficients have been reported in the literature for waves at normal incidence, hence *θ*_T_ = *θ*_L_ = 0 in (5.10). (This case does not give rise to mode conversion.) We start with this case in figure 8 and move on to oblique incidence in figure 9 (mode conversions occur). The plots show the results for four thicknesses from very thin e=0.01 to relatively thin e=0.3. It turns out that the thickness has a strong influence on the variations of the scattering coefficients, which is accurately captured by our model (dashed black lines) up to *k*_T_ *h* ∼ 2 whatever the array thinness. Next, the massless-spring model with two stiffnesses (3.28) (dashed grey lines) has roughly the same accuracy for e=0.01 and it rapidly fails in accuracy for thicker voids. Eventually the limit of a single stiffness (3.29) (providing the curve in dotted grey lines) has a limited range of validity even for the thinner array (this is more noticeable at normal incidence).

## 6. Conclusion

In this paper, we have derived effective jump conditions for an imperfect interface composed of an array of defects, e.g. voids or cracks. The conditions are expressed across an interface having the same thickness as the actual ones (and whose interior is not resolved); we have shown that this *enlarged formulation* enjoys good energetic property, as the interface has a positive energetic contribution. It is worth noticing that this property prevents from possible severe instabilities of any explicit time-discretization numerical scheme. Besides, by comparison with direct numerical calculations, we have shown that the enlarged formulation is more efficient than the *zero-thickness formulation* expressed across a zero-thickness interface. In the reported examples, we have observed that it remains efficient for any incidence and far beyond the long wavelength limit, usable almost up to the appearance of the higher modes when *k*_T_ > *β* + 2*nπ*/*h*, *n* integer. Spring models have been recovered: as a particular case, the *mass-spring model* is valid at normal incidence, and as limiting cases *spring-models* approximate our model for vanishing void thickness up to *O*(*e*/*h*).

Some extensions of the present study are straightforward, others less. Technically, the extension to three-dimensional elasticity is cumbersome but it does not present additional difficulties. The same holds if the voids are replaced by elastic inclusions with material parameters comparable to those of the matrix. The case of highly contrasted inclusions able to produce internal, low-frequency, resonances is more challenging. Already considered in the scalar, anti-plane, case by Pham *et al*. [26] and Touboul *et al*. [27], it is difficult to anticipate how the effect of these resonances translates in two- or three-dimensional elasticity. From a practical point of view, the study of guided waves by such imperfect interfaces and their link with Rayleigh, Stoneley and Love waves in realistic configurations is of interest in a geophysical context. In this spirit, the effects of the viscous fluids within the imperfect interface or the influence of non-linear contact laws, are interesting paths to explore.

## Data accessibility

The numerical simulations of the direct problem were obtained using the modal method published in Maurel & Pham [22].

## Appendix A. Positivity of the interfacial energy

We aim to show that the interfacial energy $E_{P}$ in (4.3) is definite positive. We divide the proof in two steps. In the first step, we show that

A 1 $$E_{P}\geq hF_{\text{2}2}\;{\bar{\varepsilon }}_{22}^{2}+\frac{he}{1-\varphi }\left(\frac{{\bar{\sigma }}_{11}^{2}}{\lambda +2\mu }+\frac{{\bar{\sigma }}_{12}^{2}}{\mu }\right),$$

and in the second step, we show that

A 2 $$F_{\text{2}2}=(\lambda +2\mu )e(1-\varphi )+\lambda B_{\text{1}}^{\text{2}2}+C_{\text{2}}^{\text{2}2}-\frac{\lambda^{2}}{\lambda +2\mu }(B_{\text{1}}^{\text{1}1}+e)\geq 0.$$

It follows that $E_{P}$ is positive and satisfies the inequality in (4.5). The two steps require the use of an energy minimization principle, or variational principle, expressed in terms of displacements (step 1) or in terms of stresses (step 2).

**(a) First step**

To show (1) we shall prove that

A 3 $$\forall (a,b)\in R^{2},\;(\lambda +2\mu )B_{\text{1}}^{\text{1}1}a^{2}+2(\lambda +2\mu )B_{\text{1}}^{\text{1}2}ab+\mu B_{\text{2}}^{\text{1}2}b^{2}\geq \frac{\varphi }{1-\varphi }\left((\lambda +2\mu )a^{2}+\mu b^{2}\right),$$

and

A 4 $$\text{then use }\;a=\frac{\bar{\sigma_{11}}}{\lambda +2\mu },\;b=\frac{\bar{\sigma_{12}}}{\mu }.$$

At this stage, $(a,b)\in R^{2}$ are not specified and to show (3) we consider the displacement field $Q^{ab}=aQ^{\text{1}1}+bQ^{\text{1}2}$ and its associated stress tensor $T^{ab}$. From (3.14), $\left(Q^{ab},T^{ab}\right)$ solve the problem

A 5 $$\begin{array}{rl} & {\text{div}}_{\;y}T^{ab}=0,\;T^{ab}=A(\varepsilon_{\;y}(Q^{ab})+ae_{1}{⊗}^{s}e_{1}+be_{1}{⊗}^{s}e_{2})\\ \text{and}\; & (Q^{ab},T^{ab})\;y_{2}\text{-periodic},\;T^{ab}n=0\;\text{on}\;\partial V,\;\lim_{y_{1}\to \pm \infty }\varepsilon_{\;y}(Q^{ab})=0. \end{array}\}$$

The strong formulation (5) can be derived from a weak formulation. This latter is obtained using that $\int_{Y}\text{d}\;y\;Q\cdot {\text{div}}_{\;y}T^{ab}=0$ for any Q being an admissible test function, $y_{2}$-periodic and whose gradient tends to 0 at when $y_{1}\to \pm \infty$. Integrating by parts leads to

A 6 $$\begin{array}{rl} & \int_{Y}\text{d}\;y\;\varepsilon_{\;y}(Q)\cdot A\varepsilon_{\;y}(Q^{ab})+a\int_{Y}\text{d}\;y\;\left[(\lambda +2\mu )\frac{\partial Q_{1}}{\partial y_{1}}+\lambda \frac{\partial Q_{2}}{\partial y_{2}}\right]+b\int_{Y}\text{d}\;y\;\mu \left[\frac{\partial Q_{1}}{\partial y_{2}}+\frac{\partial Q_{2}}{\partial y_{1}}\right]\\ & \;-(\lambda +2\mu )a\delta (Q_{1})-\mu b\delta (Q_{2})=0, \end{array}$$

where $\delta (Q_{k})=(\lim_{y_{1}\to +\infty }Q_{k}-\lim_{y_{1}\to -\infty }Q_{k})$, *k* = 1, 2. Such weak formulation is itself derived from a variational principle *i.e.* an energy minimization principle. In that perspective we introduce the space D of admissible displacement fields defined by

$$D=\{Q:\;Q\;y_{2}\text{-periodic},\;\text{continuous},\;\lim_{y_{1}\to \pm \infty }\varepsilon_{\;y}(Q)=0\}.$$

The potential energy $E^{ab}:D\mapsto R$ then reads

A 7 $$\begin{array}{rl} E^{ab}(Q)= & \frac{1}{2}\int_{Y}\text{d}\;y\;\varepsilon_{\;y}(Q)\cdot A\varepsilon_{\;y}(Q)+a\int_{Y}\text{d}\;y\;\left[(\lambda +2\mu )\frac{\partial Q_{1}}{\partial y_{1}}+\lambda \frac{\partial Q_{2}}{\partial y_{2}}\right]+b\int_{Y}\text{d}\;y\;\mu \left[\frac{\partial Q_{1}}{\partial y_{2}}+\frac{\partial Q_{2}}{\partial y_{1}}\right]\\ & \;-(\lambda +2\mu )a\delta (Q_{1})-\mu b\delta (Q_{2}). \end{array}$$

The variational principle is given by

A 8 $$\forall \;Q\in D,\;E^{ab}(Q)\geq E^{ab}(Q^{ab}),$$

and it can be shown that the first-order stationary condition of the potential energy $E^{ab}$ corresponds to the weak formulation (6). The potential energy of the solution is obtained taking $Q=Q^{ab}$ in (7) along with (6) and we find

$$E^{ab}(Q^{ab})=-\frac{1}{2}(\lambda +2\mu )(B_{\text{1}}^{\text{1}1}-\varphi e)a^{2}-(\lambda +2\mu )B_{\text{1}}^{\text{1}2}ab-\frac{1}{2}\mu (B_{\text{2}}^{\text{1}2}-\varphi e)b^{2},$$

where we have also used (3.23) and (3.24). Now, considering the admissible displacement test function $Q=q(y_{1})(ae_{1}+be_{2})$, with $q(y_{1}<0)=0$, $q(0<y_{1}<e)=\frac{\varphi }{1-\varphi }\;\frac{y_{1}}{e}$, and $q(y_{1}>e)=\frac{\varphi }{1-\varphi }$, we get from (7) that

A 9 $$E^{ab}(Q)=-\frac{e}{2}\frac{\varphi^{2}}{1-\varphi }\left((\lambda +2\mu )a^{2}+\mu b^{2}\right).$$

Eventually, applying the variational principle (8), it is straightforward to obtain (3). It is also straightforward to see that, with the choice of (*a*, *b*) in (4), hence $a=\bar{\varepsilon_{11}}+\frac{\lambda }{\lambda +2\mu }\bar{\varepsilon_{22}}$ and $b=2\bar{\varepsilon_{12}}$, (3) provides (1).

**(b) Second step**

We now move on the second step, where we aim to prove (2). For that we consider the specific displacement field $Q^{*}=a_{1}Q^{\text{1}1}+a_{2}Q^{\text{2}2}$ with (*a*_1_, *a*_2_) given by

A 10 $$a_{1}=-\frac{\lambda }{2(\lambda +\mu )}\;\text{and}\;a_{2}=\frac{\lambda +2\mu }{2(\lambda +\mu )}.$$

With this choice of (*a*_1_, *a*_2_), we have $A(a_{1}e_{1}{⊗}^{s}e_{1}+a_{2}e_{2}{⊗}^{s}e_{2})=2\mu e_{2}{⊗}^{s}e_{2}$. Hence, from (3.14), it is easy to check that the field $Q^{*}$ and the associated stress tensor $T^{*}$ solve

A 11 $$\begin{array}{rl} & {\text{div}}_{\;y}T^{*}=0,\;T^{*}=A\varepsilon_{\;y}(Q^{*})+2\mu e_{2}{⊗}^{s}e_{2}\\ \text{and}\; & (Q^{*},T^{*})\;y_{2}\text{-periodic},\;T^{*}n=0\;\text{on}\;\;\partial V,\;\lim_{y_{1}\to \pm \infty }\varepsilon_{\;y}(Q^{*})=0. \end{array}\}$$

We have now to make use of the variational principle in terms of the stresses (instead of the displacement as used in the first step). In this case, the space of admissible stress fields is

$$T=\left\{T:\;{\text{div}}_{\;y}T=0,T\;y_{2}\text{-periodic},\;\text{continuous},\;Tn=0\;\text{on}\;\partial V,\lim_{y_{1}^{\text{m}}\to \pm \infty }T=2\mu e_{2}{⊗}^{s}e_{2}\right\}.$$

It can be shown that the problem (11) is formally equivalent to the variational principle

A 12 $$\forall \;T\in T,\;E^{*}(T)\geq E^{*}(T^{*}).$$

where the complementary potential energy $E^{*}:T\mapsto R$ is given by

A 13 $$E^{*}(T)=\frac{1}{2}\int_{\Omega }\text{d}\;y\;(T-2\mu e_{2}{⊗}^{s}e_{2})\cdot A^{-1}(T-2\mu e_{2}{⊗}^{s}e_{2}).$$

We start by evaluate $E^{*}$ at its minimum $T^{*}$ which reads $E^{*}(T^{*})=\frac{1}{2}\int_{Y}\text{d}\;y\;\varepsilon_{\;y}(Q^{*})\cdot A\varepsilon_{\;y}(Q^{*})$. With $q_{ijkl}=\int_{Y}\text{d}\;y\;\varepsilon_{\;y}(Q^{kl})\cdot A\varepsilon_{\;y}(Q^{ij})$ introduced in (3.25), we have $E^{*}(T^{*})=\frac{1}{2}(q_{\text{1}111}a_{1}^{2}+2q_{\text{1}122}a_{1}a_{2}+q_{\text{2}222}a_{2}^{2})$, and making use of (3.24), we eventually get

A 14 $$E^{*}(T^{*})=(\lambda +2\mu )(B_{\text{1}}^{\text{1}1}-\varphi e)\frac{a_{1}^{2}}{2}+\left((\lambda +2\mu )B_{\text{1}}^{\text{2}2}-\lambda \varphi e\right)a_{1}a_{2}+(\lambda B_{\text{1}}^{\text{2}2}-C_{\text{2}}^{\text{2}2})\frac{a_{2}^{2}}{2},$$

(remember that we have defined $C_{\text{2}}^{\text{2}2}=D_{\text{2}2}^{\text{2}2}$). Now we consider the admissible stress test function $T=t(y_{1})\;e_{2}{⊗}^{s}e_{2}$ with $t(0<y_{1}<e)=0$, and $t(y_{1})=2\mu$ otherwise. By construction we have that $A^{-1}(2\mu e_{2}{⊗}^{s}e_{2})=a_{1}e_{1}{⊗}^{s}e_{1}+a_{2}e_{2}{⊗}^{s}e_{2}$, hence the energy (13) of this test function reads

$$E^{*}(T)=\mu e(1-\varphi )a_{2}.$$

Applying the variational principle (8) along with (*a*_1_, *a*_2_) in (10) leads to (2).

## Appendix B. Scattering coefficients

In this appendix and for compactness, we use ${\text{s}}_{\theta }=\sin\theta$ and ${\text{c}}_{\theta }=\cos\theta$ and $\left(C_{\text{2}}^{\text{1}1},C_{\text{2}}^{\text{2}2}\right)$ stand for $\left(C_{\text{2}}^{\text{1}1}/\mu ,C_{\text{2}}^{\text{2}2}/\mu \right)$ We also define $\xi =\sqrt{\lambda +2\mu /\mu }$.

By linearity the system can be solved separately for an incident transverse/longitudinal wave. We consider first an incident transverse wave with *A*_L_ = 0, *A*_T_ = 1. From (5.3) and with $u=\nabla \Phi +\nabla \times (\Psi e_{3})$ and $\beta =k_{\text{L}}{\text{s}}_{\theta_{\text{L}}}=k_{\text{T}}{\text{s}}_{\theta_{\text{T}}}$, we have

B 1 $$\left\{ \begin{array}{ll} u_{1}(0,x_{2})=\text{i}k_{\text{L}}[-{\text{c}}_{\theta_{\text{L}}}r_{\text{L}T}+{\text{s}}_{\theta_{\text{L}}}(1+r_{\text{T}T})]{\text{e}}^{\text{i}\beta x_{2}},\; & u_{2}(0,x_{2})=\text{i}k_{\text{T}}[{\text{s}}_{\theta_{\text{T}}}r_{\text{L}T}-{\text{c}}_{\theta_{\text{T}}}(1-r_{\text{T}T})]{\text{e}}^{\text{i}\beta x_{2}},\\ u_{1}(e,x_{2})=\text{i}k_{\text{L}}[{\text{c}}_{\theta_{\text{L}}}t_{\text{L}T}+{\text{s}}_{\theta_{\text{L}}}t_{\text{T}T}]{\text{e}}^{\text{i}\beta x_{2}}, & u_{2}(e,x_{2})=\text{i}k_{\text{T}}[{\text{s}}_{\theta_{\text{T}}}t_{\text{L}T}-{\text{c}}_{\theta_{\text{T}}}t_{\text{T}T}]{\text{e}}^{\text{i}\beta x_{2}}, \end{array}\right.$$

and all the quantities needed in (5.1) can be deduced from the displacements. It turns out that the four jump conditions are decoupled: the relations ([[*u*_1_]], [[*σ*_12_]]) involve *S*_LT_ = (*r*_LT_ + *t*_LT_) and *D*_TT_ = (*t*_TT_ − *r*_TT_) while ([[*u*_2_]], [[*σ*_11_]]) involve *D*_LT_ = (*t*_LT_ − *r*_LT_) and *S*_TT_ = (*t*_TT_ + *r*_TT_). The systems reads

B 2 $$\left(\begin{array}{ll} a_{1} & b_{1}\\ c_{1} & d_{1} \end{array}\right)\left(\begin{array}{l} S_{\text{L}T}\\ D_{\text{T}T} \end{array}\right)=\left(\begin{array}{l} b_{1}^{*}\\ d_{1}^{*} \end{array}\right),\;\left(\begin{array}{ll} a_{2} & b_{2}\\ c_{2} & d_{2} \end{array}\right)\left(\begin{array}{l} D_{\text{L}T}\\ S_{\text{T}T} \end{array}\right)=\left(\begin{array}{l} b_{2}^{*}\\ d_{2}^{*} \end{array}\right),$$

where * means complex conjugate and with

B 3 $$\begin{array}{rl} & a_{1}={\text{c}}_{\theta_{\text{L}}}-\frac{\text{i}k_{\text{L}}h}{2}[{\text{c}}_{\theta_{\text{L}}}^{2}(e+B_{\text{1}}^{\text{1}1})+{\text{s}}_{\theta_{\text{L}}}^{2}B_{\text{1}}^{\text{2}2})],\;b_{1}={\text{s}}_{\theta_{\text{L}}}\left[1-\frac{\text{i}k_{\text{T}}h}{2}{\text{c}}_{\theta_{\text{T}}}(e+B_{\text{1}}^{\text{1}1}-B_{\text{1}}^{\text{2}2})\right],\\ & c_{1}={\text{s}}_{2\theta_{\text{L}}}-\frac{\text{i}k_{\text{L}}h}{2}{\text{s}}_{\theta_{\text{L}}}[2{\text{c}}_{\theta_{\text{L}}}^{2}(1-\varphi )e-{\text{c}}_{\theta_{\text{L}}}^{2}C_{\text{2}}^{\text{1}1}-{\text{s}}_{\theta_{\text{L}}}^{2}C_{\text{2}}^{\text{2}2}],\\ & d_{1}=\xi^{2}\left[-{\text{c}}_{2\theta_{\text{T}}}+\frac{\text{i}k_{\text{T}}h}{2}{\text{c}}_{\theta_{\text{T}}}\left({\text{c}}_{2\theta_{\text{T}}}(1-\varphi )e+{\text{s}}_{\theta_{\text{T}}}^{2}(C_{\text{2}}^{\text{1}1}-C_{\text{2}}^{\text{2}2})\right)\right],\\ & a_{2}=-{\text{s}}_{\theta_{\text{T}}}+\frac{\text{i}k_{\text{L}}h}{2}{\text{s}}_{\theta_{\text{T}}}{\text{c}}_{\theta_{\text{L}}}\left(e+2B_{\text{2}}^{\text{1}2}\right),\;b_{2}={\text{c}}_{\theta_{\text{T}}}-\frac{\text{i}k_{\text{T}}h}{2}\left({\text{c}}_{\theta_{\text{T}}}^{2}e+{\text{c}}_{2\theta_{\text{T}}}B_{\text{2}}^{\text{1}2}\right),\\ & c_{2}={\text{c}}_{2\theta_{\text{T}}}-\frac{\text{i}k_{\text{L}}h}{2}{\text{c}}_{\theta_{\text{L}}}\left({\text{c}}_{2\theta_{\text{T}}}-\varphi \right)e,\;d_{2}={\text{s}}_{2\theta_{\text{T}}}+\frac{\text{i}k_{\text{L}}h}{2}{\text{s}}_{\theta_{\text{L}}}\left(\varphi -2{\text{c}}_{\theta_{\text{T}}}^{2}\right)e. \end{array}\}$$

Now we consider an incident longitudinal wave (*A*_L_ = 1, *A*_T_ = 0). We have

B 4 $$\begin{array}{rl} & u_{1}(0,x_{2})=\text{i}(\alpha_{\text{L}}(1-r_{\text{L}L})+\beta r_{\text{T}L}){\text{e}}^{\text{i}\beta x_{2}},\;u_{2}(0,x_{2})=\text{i}(\beta (1+r_{\text{L}L})+\alpha_{\text{T}}r_{\text{T}L}){\text{e}}^{\text{i}\beta x_{2}},\\ \;\text{and}\; & u_{1}(e,x_{2})=\text{i}(\alpha_{\text{L}}t_{\text{L}L}+\beta t_{\text{T}L}){\text{e}}^{\text{i}\beta x_{2}},\;u_{2}(0,x_{2})=\text{i}(\beta t_{\text{L}L}-\alpha_{\text{T}}t_{\text{T}L})){\text{e}}^{\text{i}\beta x_{2}}, \end{array}\}$$

resulting in two decoupled systems on *S*_LL_ = (*r*_LL_ + *t*_LL_), *D*_TL_ = (*t*_TL_ − *r*_TL_) and on *D*_LL_ = (*t*_LL_ − *r*_LL_), *S*_TL_ = (*t*_TL_ + *r*_TL_) of the form

B 5 $$\left(\begin{array}{ll} a_{1} & b_{1}\\ c_{1} & d_{1} \end{array}\right)\left(\begin{array}{l} S_{\text{L}L}\\ D_{\text{T}L} \end{array}\right)=\left(\begin{array}{l} a_{1}^{*}\\ c_{1}^{*} \end{array}\right),\;\left(\begin{array}{ll} a_{2} & b_{2}\\ c_{2} & d_{2} \end{array}\right)\left(\begin{array}{l} D_{\text{L}L}\\ S_{\text{T}L} \end{array}\right)=\left(\begin{array}{l} a_{2}^{*}\\ c_{2}^{*} \end{array}\right).$$

The two systems can be inverted which provides explicit expressions

B 6 $$\begin{array}{rl} & r_{\text{L}L}=\frac{1}{2}(S_{\text{L}L}-D_{\text{L}L}),\;r_{\text{T}T}=\frac{1}{2}(S_{\text{T}T}-D_{\text{T}T}),\;r_{\text{L}T}=\frac{1}{2}(S_{\text{L}T}-D_{\text{L}T}),\;r_{\text{T}L}=\frac{1}{2}(S_{\text{T}L}-D_{\text{T}L})\\ \text{and}\; & t_{\text{L}L}=\frac{1}{2}(S_{\text{L}L}+D_{\text{L}L}),\;t_{\text{T}T}=\frac{1}{2}(S_{\text{T}T}+D_{\text{T}T}),\;t_{\text{L}T}=\frac{1}{2}(S_{\text{L}T}+D_{\text{L}T}),\;t_{\text{T}L}=\frac{1}{2}(S_{\text{T}L}+D_{\text{T}L}), \end{array}\}$$

with

B 7 $$\begin{array}{rl} & D_{\text{L}L}=\frac{1}{\Delta_{\text{L}}}\left[1+\frac{{\left(k_{\text{T}}h\right)}^{2}}{4\xi }\frac{{\text{c}}_{\theta_{\text{L}}}}{{\text{c}}_{\theta_{\text{T}}}}e\left[(1-\varphi )(e+B_{\text{1}}^{\text{1}2})-{\text{s}}_{\theta_{\text{T}}}^{2}e\right]+\frac{\text{i}k_{\text{T}}h}{2\xi }\left[\frac{f_{\text{θ}}^{-}}{{\text{c}}_{\theta_{\text{T}}}}B_{\text{1}}^{\text{1}2}+\frac{{\text{s}}_{\left(\theta_{\text{T}}-\theta_{\text{L}}\right)}}{{\text{s}}_{\theta_{\text{T}}}}e-\frac{{\text{c}}_{\left(\theta_{\text{L}}+\theta_{\text{T}})\right)}}{{\text{c}}_{\theta_{\text{T}}}}\varphi e\right]\right],\\ & S_{\text{T}T}=\frac{\Delta_{\text{L}}^{*}}{\Delta_{\text{L}}}D_{\text{L}L}^{*},\;S_{\text{T}L}=\frac{\text{i}k_{\text{T}}h}{\Delta_{\text{L}}\xi }\frac{{\text{c}}_{\theta_{\text{L}}}}{{\text{c}}_{\theta_{\text{T}}}}\;{\text{s}}_{\theta_{\text{T}}}[\varphi e+2{\text{c}}_{2\theta_{\text{T}}}B_{\text{1}}^{\text{1}2}],\;D_{\text{L}T}=-\frac{\text{i}k_{\text{T}}h}{\Delta_{\text{L}}}{\text{s}}_{\theta_{\text{T}}}[\varphi e+2{\text{c}}_{2\theta_{\text{T}}}B_{\text{1}}^{\text{1}2}],\\ & \text{with }\;\Delta_{\text{L}}=1-\frac{{\left(k_{\text{T}}h\right)}^{2}}{4\xi }\frac{{\text{c}}_{\theta_{\text{L}}}}{{\text{c}}_{\theta_{\text{T}}}}e\left[(1-\varphi )(e+B_{\text{1}}^{\text{1}2})-{\text{s}}_{\theta_{\text{T}}}^{2}e\right]-\frac{\text{i}k_{\text{T}}h}{2\xi }\left[\frac{f_{\theta }^{+}}{{\text{c}}_{\theta_{\text{T}}}}B_{\text{1}}^{\text{1}2}+\frac{{\text{s}}_{\left(\theta_{\text{T}}+\theta_{\text{L}}\right)}}{{\text{s}}_{\theta_{\text{T}}}}e-\frac{{\text{c}}_{\left(\theta_{\text{L}}-\theta_{\text{T}}\right)}}{{\text{c}}_{\theta_{\text{T}}}}\varphi e\right],\\ & \;f_{\theta }^{-}=\frac{1}{\xi }{\text{s}}_{2\theta_{\text{L}}}{\text{s}}_{2\theta_{\text{T}}}-\xi {\text{c}}_{2\theta_{\text{T}}}^{2},\;f_{\theta }^{+}=\frac{1}{\xi }{\text{s}}_{2\theta_{\text{L}}}{\text{s}}_{2\theta_{\text{T}}}+\xi {\text{c}}_{2\theta_{\text{T}}}^{2} \end{array}\}$$

and

B 8 $$\begin{array}{c} S_{\mathrm{LL}}=\frac{1}{\Delta_{T}}[1+\frac{{\left(k_{T}h\right)}^{2}}{4\xi }\frac{c_{\theta_{T}}}{c_{\theta_{L}}}\left[\left(c_{\theta_{L}}^{2}(1-\varphi )e-s_{\theta_{T}}^{2}\left((2-\xi^{2})B_{1}^{22}+C_{2}^{22}\right)\right)(e+B_{1}^{11})-s_{\theta_{L}}^{2}{\left(B_{1}^{22}\right)}^{2}\right]\;\\ -\frac{ik_{T}h}{2}\left[\frac{c_{\left(\theta_{L}+\theta_{T}\right)}}{c_{\theta_{L}}}\left(c_{\theta_{L}}^{2}(1-\varphi )e-s_{\theta_{T}}^{2}C_{2}^{22}\right)+\left(c_{\theta_{T}}s_{\theta_{L}}^{2}-\frac{c_{\theta_{L}}^{3}}{\xi }\right)(e+B_{1}^{11})-g_{\theta }^{+}B_{1}^{22}\right]],\;\\ D_{\mathrm{TT}}=\frac{\Delta_{T}^{*}}{\Delta_{T}}S_{\mathrm{LL}}^{*},\;S_{\mathrm{LT}}=\frac{ik_{T}h}{\Delta_{T}}\frac{c_{\theta_{T}}}{c_{\theta_{L}}}\;s_{\theta_{L}}[c_{\theta_{L}}^{2}(B_{1}^{11}-B_{1}^{22}+\varphi e)+s_{\theta_{T}}^{2}(2B_{1}^{22}+C_{2}^{22})],\;\\ D_{\mathrm{TL}}=\frac{ik_{T}h}{\Delta_{T}}\;s_{\theta_{T}}[c_{\theta_{L}}^{2}(B_{1}^{11}-B_{1}^{22}+\varphi e)+s_{\theta_{T}}^{2}(2B_{1}^{22}+C_{2}^{22})],\;\\ \mathrm{with}\;\\ \;\Delta_{T}=-\frac{{\left(k_{T}h\right)}^{2}}{4\xi }\frac{c_{\theta_{T}}}{c_{\theta_{L}}}\left[\left(c_{\theta_{L}}^{2}(1-\varphi )e-s_{\theta_{T}}^{2}\left((2-\xi^{2})B_{1}^{22}+C_{2}^{22}\right)\right)(e+B_{1}^{11})-s_{\theta_{L}}^{2}{\left(B_{1}^{22}\right)}^{2}\right]\;\\ \;-\frac{ik_{T}h}{2}\left[\frac{c_{\left(\theta_{L}-\theta_{T}\right)}}{c_{\theta_{L}}}\left(c_{\theta_{L}}^{2}(1-\varphi )e-s_{\theta_{T}}^{2}C_{2}^{22}\right)+\left(c_{\theta_{T}}s_{\theta_{L}}^{2}+\frac{c_{\theta_{L}}^{3}}{\xi }\right)(e+B_{1}^{11})-g_{\theta }^{-}B_{1}^{22}\right],\;\\ g_{\theta }^{+}=\frac{s_{\theta_{L}}^{2}}{\xi }\left[\left(\frac{2}{\xi }+\xi \right)c_{\theta_{T}}+\frac{c_{2\theta_{T}}}{c_{\theta_{L}}}+c_{\theta_{L}}\right],\;g_{\theta }^{-}=\frac{s_{\theta_{L}}^{2}}{\xi }\left[\left(\frac{2}{\xi }+\xi \right)c_{\theta_{T}}-\frac{c_{2\theta_{T}}}{c_{\theta_{L}}}-c_{\theta_{L}}\right].\; \end{array}\}$$

Deep simplifications occur when the void thickness e vanishes, resulting in C→0. Specifically, with the definitions of F, G, *d*_F_, *d*_G_ in (5.9), we have

B 9 $$\begin{array}{rl} & S_{\text{L}L}=\frac{1}{d_{\text{F}}}\left[{\text{s}}_{2\theta_{\text{L}}}{\text{s}}_{2\theta_{\text{T}}}-\xi^{2}{\text{c}}_{2\theta_{\text{T}}}^{2}+\text{i}\text{F}{\text{c}}_{\theta_{\text{L}}}\right],\;D_{\text{L}L}=-\frac{1}{d_{\text{G}}}\left[{\text{s}}_{2\theta_{\text{L}}}{\text{s}}_{2\theta_{\text{T}}}-\xi^{2}{\text{c}}_{2\theta_{\text{T}}}^{2}-\text{i}\text{G}{\text{c}}_{\theta_{\text{T}}}\right],\\ & S_{\text{T}T}=\frac{1}{d_{\text{G}}}\left[{\text{s}}_{2\theta_{\text{L}}}{\text{s}}_{2\theta_{\text{T}}}-\xi^{2}{\text{c}}_{2\theta_{\text{T}}}^{2}+\text{i}\text{G}{\text{c}}_{\theta_{\text{T}}}\right],\;D_{\text{T}T}=-\frac{1}{d_{\text{F}}}\left[{\text{s}}_{2\theta_{\text{L}}}{\text{s}}_{2\theta_{\text{T}}}-\xi^{2}{\text{c}}_{2\theta_{\text{T}}}^{2}-\text{i}\text{F}{\text{c}}_{\theta_{\text{L}}}\right],\\ & S_{\text{T}L}=\frac{2{\text{s}}_{2\theta_{\text{L}}}{\text{c}}_{2\theta_{\text{T}}}}{d_{\text{G}}},\;D_{\text{T}L}=-\frac{2{\text{s}}_{2\theta_{\text{L}}}{\text{c}}_{2\theta_{\text{T}}}}{d_{\text{F}}},\;S_{\text{L}T}=-\frac{2\xi^{2}{\text{s}}_{2\theta_{\text{T}}}{\text{c}}_{2\theta_{\text{T}}}}{d_{\text{F}}},\;D_{\text{L}T}=\frac{2\xi^{2}{\text{c}}_{2\theta_{\text{T}}}{\text{s}}_{2\theta_{\text{T}}}}{d_{\text{G}}}. \end{array}\}$$
